# The digestive tract as an essential organ for water acquisition in marine teleosts: lessons from euryhaline eels

**DOI:** 10.1186/s40851-021-00175-x

**Published:** 2021-06-21

**Authors:** Yoshio Takei

**Affiliations:** Laboratory of Physiology, Department of Marine Bioscience, Atmosphere and Ocean Research Institute, The University of Tokyo, 5-1-5 Kashiwanoha, Kashiwa, Chiba, 277-8564 Japan

**Keywords:** Osmoregulation, Seawater adaptation, *Anguilla* spp., Esophageal desalinization, Biomineralization, Epithelial transport, Transcellular transport, Paracellular transport, Tight junction protein, Hormonal regulation, Metabolon, Vesicle trafficking

## Abstract

Adaptation to a hypertonic marine environment is one of the major topics in animal physiology research. Marine teleosts lose water osmotically from the gills and compensate for this loss by drinking surrounding seawater and absorbing water from the intestine. This situation is in contrast to that in mammals, which experience a net osmotic loss of water after drinking seawater. Water absorption in fishes is made possible by (1) removal of monovalent ions (desalinization) by the esophagus, (2) removal of divalent ions as carbonate (Mg/CaCO_3_) precipitates promoted by HCO_3_^−^ secretion, and (3) facilitation of NaCl and water absorption from diluted seawater by the intestine using a suite of unique transporters. As a result, 70–85% of ingested seawater is absorbed during its passage through the digestive tract. Thus, the digestive tract is an essential organ for marine teleost survival in the hypertonic seawater environment. The eel is a species that has been frequently used for osmoregulation research in laboratories worldwide. The eel possesses many advantages as an experimental animal for osmoregulation studies, one of which is its outstanding euryhalinity, which enables researchers to examine changes in the structure and function of the digestive tract after direct transfer from freshwater to seawater. In recent years, the molecular mechanisms of ion and water transport across epithelial cells (the transcellular route) and through tight junctions (the paracellular route) have been elucidated for the esophagus and intestine. Thanks to the rapid progress in analytical methods for genome databases on teleosts, including the eel, the molecular identities of transporters, channels, pumps and junctional proteins have been clarified at the isoform level. As 10 y have passed since the previous reviews on this subject, it seems relevant and timely to summarize recent progress in research on the molecular mechanisms of water and ion transport in the digestive tract in eels and to compare the mechanisms with those of other teleosts and mammals from comparative and evolutionary viewpoints. We also propose future directions for this research field to achieve integrative understanding of the role of the digestive tract in adaptation to seawater with regard to pathways/mechanisms including the paracellular route, divalent ion absorption, metabolon formation and cellular trafficking of transporters. Notably, some of these have already attracted practical attention in laboratories.

**Supplementary Information:**

The online version contains supplementary material available at 10.1186/s40851-021-00175-x.

## 1. Background

### 1.1 Diverse body fluid regulation in marine fishes

**Fig. 1:**
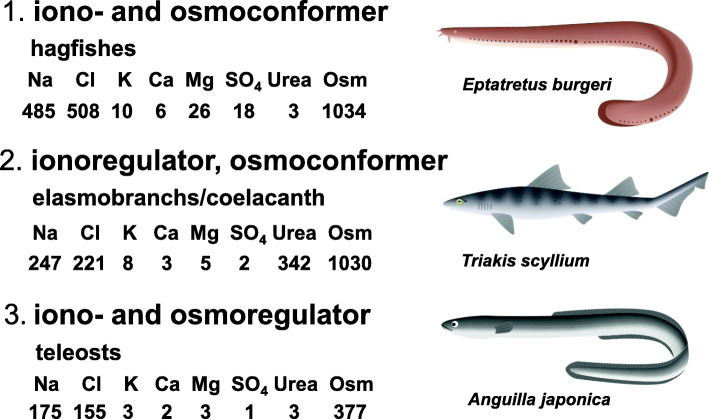
Diverse strategies for adaptation of fishes to a hypertonic seawater environment. The ion concentrations are in mM, and osmolality (Osm) is in mOsm/l. The values for eels are for eels acclimated to seawater. The values for seawater are shown in Table 1. All marine mammals, birds and reptiles are iono- and osmoregulators, while the only amphibian that can acclimate to seawater, the crab-eating frog (*Fejervarya cancrivora*), is an ionoregulator and osmoconformer, as are elasmobranchs. Illustrated by Mari Kawaguchi of Sophia University (https://www.ginganet.org/mari)

Extant vertebrates inhabit three different types of habitats in terms of osmoregulation: water- and ion-deficient land habitats, water-sufficient and ion-deficient inland freshwater (FW) habitats, and water- and ion-sufficient seawater (SW) habitats. Because of the high osmolality of SW, however, the ocean is generally a desiccative environment for marine teleost fishes. To address this issue, marine fishes use three different strategies for body fluid regulation (Fig. 1). The first strategy is adopted by the most primitive extant vertebrate group, the hagfishes, which conform their plasma to environmental SW in terms of both ion concentrations and osmolality (acting as iono- and osmoconformers), similar to marine invertebrates (Fig. 1). Thus, they exert little osmoregulation effort and incur little expense. The ions involved are monovalent ions (Na^+^ and Cl^−^), as divalent ions (Mg^2+^ and SO_4_^2−^) are maintained at levels lower than those in plasma [63]. The second strategy is employed by elasmobranchs and a lobe-finned bony fish, the coelacanth, whose plasma ion concentrations are lower than the concentrations in SW but whose plasma osmolality is maintained at a level similar to that in SW via accumulation of urea in the plasma (acting as ionoregulators and osmoconformers). Thus, they need only to excrete excess ions and retain urea at the kidneys, as osmotic forces on water diffusion are nearly abolished. Marine teleosts use a third strategy like that of marine mammals, birds and reptiles, which have plasma ion concentrations and osmolality approximately one-third that of SW (making them iono- and osmoregulators) (Fig. 1). Thus, they must counter osmotic water loss and excess ion gain. To address this hydromineral challenge, marine teleosts drink SW and absorb water together with ions in the intestine. Ions also enter the body via the body surfaces driven by their concentration gradients. Then, excess monovalent ions are excreted mostly by the gills, and excess divalent ions are excreted by the kidneys [127, 144].

Gill ionocytes (mitochondrion-rich cells) are responsible for active Na^+^ and Cl^−^ excretion (see [36, 51]). As acquisition of ionocytes appeared to be one of the cues for teleosts to re-enter the marine environment after the teleost-specific whole-genome duplication that occurred ca. 300 mya [91], gill ionocytes have been the most intensively studied tissue in osmoregulation research. Consequently, the molecular mechanisms of transcellular and paracellular ion transport have been elucidated, and these mechanisms have been summarized in several reviews (e.g., [30, 85, 86, 90]). SW contains high concentrations of the divalent ions Mg^2+^ and SO_4_^2−^ in addition to monovalent ions (Table 1), and the sites of MgSO_4_ excretion are the proximal tubules of the kidneys [18, 19]. Transporters involved in transcellular Mg^2+^ transport have been suggested to exist in the euryhaline pufferfish mefugu, *Takifugu obscurus* [96, 97], and those for SO_4_^2−^ transport have been proposed to exist in the mefugu [107] and eel [256]. However, the whole picture of divalent ion transport in the kidneys has not yet been revealed. The urinary bladder also serves as a site for final water absorption before excretion in marine teleosts [134].

**Table 1:** Ion concentrations and osmolality of luminal fluid along the digestive tract of eel. Ion concentrations of eel plasma and of seawater are also shown

Segment	Osmolality (mOsm/l)	Na^+^ (mM)	*K^+^ (mM)	Cl^-^ (mM)	HCO_3_^-^ (mM)	Mg^2+^ (mM)	Ca^2+^ (mM)	SO_4_^2-^ (mM)
Esophagus	449	211	10	225	-	40	8	-
Stomach	460	222	14	255	-	41	10	-
Intestine	295	40	14	68	100	150	12	133
Rectum	300	4	11	59	105	188	14	105
SW	1029	450	10	524	2	50	10	30
Plasma	377	175	4	155	14	3	2	1

-, not measured. The data are based on Tsukada et al. [241]

*Data of *Pleuronectes platessa* [74]

It has been known since the early 1900s that marine teleosts drink copiously to compensate for water lost osmotically across the body surface [207]. The mechanisms for eliciting drinking have been investigated in various teleost species and have been summarized in a few reviews [69, 218, 221, 259]. Relatively recently, the roles of cerebral mechanisms regulating drinking (thirst) in teleosts were suggested after the mechanisms were compared between fully aquatic eels and semiterrestrial mudskippers [11, 105]. After SW is consumed, it enters the digestive tract, which is an intracorporeal but external environment in relation to body fluids. Only after absorption by the intestine does the ingested water join the body fluids. In this sense, intestinal water absorption is critical for body fluid regulation and thus for survival of marine teleosts in hypertonic SW.

### 1.2 Role of the digestive tract in adaptation to marine environments

For terrestrial vertebrates such as mammals, drinking SW results in severe loss of body fluids. Water is lost by osmosis in the esophagus and stomach when hypertonic SW enters the lumen. NaCl is absorbed significantly by the intestine and NaCl absorption may be accompanied by absorption of some water. However, as Mg^2+^ and SO_4_^2−^ in SW are scarcely absorbed by the intestine, the luminal fluid osmolality increases after water absorption, which hinders further water absorption. Thus, severe diarrhea occurs after SW drinking in mammals. Moreover, as urine NaCl concentrations of terrestrial mammals are usually lower than SW NaCl concentrations, water is further lost in the urine for NaCl excretion. Although there is a report showing that whale kidneys can concentrate Cl^−^ to a concentration of 820 mM [189], urine NaCl concentrations are usually much lower than SW concentrations [20]. Marine birds and reptiles possess salt glands that concentrate NaCl above SW levels through a mechanism similar to that in teleost ionocytes, which are localized in different parts of the body [189].

On the other hand, marine teleosts can absorb 70–85% of ingested SW across the intestine and excrete excess NaCl from gill ionocytes [127, 144]. This is due to the excellent ability of the teleost digestive tract to process ingested SW along its segments for final absorption in the intestine (see [69, 259]). As detailed below, ingested SW is first diluted in the esophagus by removal of Na^+^ and Cl^−^ (desalinization) without water loss [83, 166]. The ingested SW is further diluted in the anterior intestine by bicarbonate ion (HCO_3_^−^) secretion into the lumen, which is followed by reductions in Mg^2+^ and Ca^2+^ concentrations via precipitation of these ions as carbonates [70, 73, 264]. Finally, water is absorbed in parallel with Na^+^ and Cl^−^ by the intestine, either transcellularly via the suite of transporters unique to marine teleosts [69, 133, 259] or paracellularly via tight junctions (TJs).

Thanks to the recent development of gene technology and bioinformatics together with the establishment of genome databases for various fish species, it is possible to identify transport molecules responsible for ion and water absorption along the digestive tract. The progress made in this decade is evident: molecular mechanisms have been elucidated at the isoform (paralog) level for transporters, channels, pumps and TJ proteins. In response to such progress and because almost 10 y have passed since the previous reviews on teleost intestinal function for osmoregulation were published [69, 213, 259], we decided to summarize current knowledge about the molecular mechanisms in order to gain insights into the future directions. In this review, we focus on the recently identified transport molecules in relation to the specific function of each segment of the digestive tract. Although molecular studies have been performed in several teleost species, we describe the mechanisms mainly in eels (genus *Anguilla*) for reasons explained in the next section. We include data obtained in mammals wherever appropriate to compare the mechanisms of water-retaining and ion-excreting marine teleosts with those of water- and ion-retaining mammals [225].

### 1.3 Contribution of eels to osmoregulation research

Among eels in the order Anguilliformes, the eel discussed here is a euryhaline, migratory (catadromous) species that can readily adapt to both hypotonic and hypertonic environments. Eels are SW species in origin, and some eels do not undergo upstream migration but rather stay in coastal SW areas throughout their lives, as shown by the presence of strontium in whole otoliths [242]. Thus, they have an excellent hypo-osmoregulation ability and can easily survive in concentrated SW. Accordingly, they can be used to examine how osmoregulatory mechanisms are altered after direct transfer from FW to SW seemingly without severe disturbances in other homeostatic mechanisms. For these reasons, the European eel (*A. anguilla*), Japanese eel (*A. japonica*) and American eel (*A. rostrata*) have often been used for osmoregulation researches since the time of Smith [207]. These researches include research on gill function [100, 103, 108, 186], drinking regulation [82, 141, 161, 223], and digestive tract function [5, 83, 114, 138, 165, 206, 238]. In contrast, another family of euryhaline migratory (anadromous) species, the salmonids, are FW species in origin, and some live their whole lives in FW (land-locked subspecies). As a result, the osmoregulatory mechanisms of eels differ considerably from those of salmonids and from those of stenohaline sedentary marine teleosts [224]. Thus, it is worthwhile to compare the roles of the digestive tract in SW adaptation among the three groups with different osmoregulatory mechanisms.

Another advantage of the eel as an experimental fish for studying intestinal function is that this fish can survive normally for several months without food. We acclimated eels in FW aquaria for 1 week after purchase and then transferred them to SW aquaria for 2 weeks to prepare SW-acclimated eels. We used them for intestinal experiments thereafter, and we obtained a full response to hormones in terms of sensitivity and efficacy for a few months thereafter (see Sect. 5), although in killifish (*Fundulus heteroclitus*), the water permeability of the intestine appears to decrease within 24 h after feeding [274]. The major function of the intestine is nutrient absorption, and ions and water in the food and in the digestive juice secreted after feeding significantly influence salt and water balance in fish [273]. We can exclude such influences when eels are used as experimental fish. In addition, we can exclude the influences of nutrient-coupled ion absorption on water and ion balance when unfed eels are used. It is known that ~ 50% of water is absorbed in parallel with Na^+^-glucose cotransport in the human intestine [132]. Furthermore, whole-genome sequencing of three *Anguill*a species has been completed, and the results are publicly available [79, 101, 168]. Thus, mining of transporter genes at the paralog level is possible with the database. In this review, data for eels are primarily introduced with particular emphasis on the molecular mechanism to serve as a basis for comparison with the molecular mechanisms of other teleosts. This review updates the previous reviews on the role of the intestine in osmoregulation [69, 213, 259] and extends our previous review on the regulation of drinking in fishes, i.e., ingestion from the environment into the digestive tract [220]. Ion transport by the digestive tract significantly affects acid-base balance, which is referred to only when necessary in this review. Readers can find several reviews on this topic elsewhere [76, 232, 272]. A detailed account of osmoregulation in invertebrates and vertebrates can be found in Larsen et al. [127].

## 2. The esophagus as an organ for desalinization

The class *Teleostei* is the most diverse group of vertebrates in terms of ecology and physiology and contains more than half of the total number of vertebrate species [159]. Unsurprisingly, the morphology of the digestive tract is also diverse among teleost species [261]. Most teleost digestive tracts include an esophagus, stomach, intestine, and rectum; some lack the stomach, such as that of medaka (*Oryzias* spp.), and some have pyloric caeca at the anterior part of the intestine, as observed in salmonids. The esophagus is the first segment of the digestive tract that directly receives ingested SW. There are sphincters at the entrance and exit of the esophagus to regulate inflow from the buccal cavity and outflow to the stomach. Therefore, ingested SW is kept in the esophagus for some time to allow modifications to its composition. The eel is an excellent experimental fish with which to study esophageal function because this fish has a long and distensible esophagus that is suitable for use in sac experiments [83, 114, 157, 227]. A morphological study has shown that the eel esophageal epithelium becomes thinner and that blood vessels develop beneath the epithelium after SW acclimation, supporting the occurrence of facilitated desalinization [275].

As shown in Table 1, ingested SW is processed gradually according to its passage through the digestive tract of eels [113, 207, 241, 257]. Similar results have been reported in the digestive tract of the winter flounder, *Pseudopleuronectes americanus* [166]. In both species, profound decreases in Na^+^ and Cl^−^ concentrations (desalinization) occur in the esophagus. In contrast, the decreases in Mg^2+^ and SO_4_^2−^ concentrations are small, suggesting that esophageal epithelia are scarcely permeable to divalent ions and water; thus, osmotic influx of water from the serosal side is negligible [83]. In vitro studies using esophageal sac preparation have shown that NaCl efflux increases while water influx decreases dramatically after SW acclimation in eels when SW is on the luminal side and isotonic Ringer solution is on the serosal side [83, 227]. Unidirectional (mucosa-to-serosa) ^22^Na fluxes have also been examined using esophageal epithelia in Ussing chambers for the winter flounder [166] and the gulf toadfish, *Opsanus beta* [49]; the results show that ^22^Na efflux is elevated in fish kept in hypersaline media.

The transporters involved in transepithelial Na^+^ and Cl^−^ transport have been examined using various transporter-specific inhibitors (Table 2). Although the efficacy of inhibition varies among species, consistent effects are obtained with mucosal application of DMA/EIPA and DIDS/DNDS and with serosal application of ouabain and DPC, suggesting the involvement of the apical Na^+^/H^+^ exchanger (NHE) and Cl^−^/HCO_3_^−^ exchanger (anion exchanger, AE) and of basolateral Na^+^/K^+^-ATPase (NKA) and Cl^−^ channels (ClCs) [49, 157, 166, 227]. HCTZ is effective in the eel [227] but not effective in the toadfish [49]. We initially thought that HCTZ inhibits the SLC12 family of transporters, such as the Na^+^-Cl^−^ cotransporter (NCC), found in the intestine (see 4.4.1), but HCTZ also inhibits carbonic anhydrase (CA), which supplies H^+^ and HCO_3_^−^ for NHE and AE to facilitate their combined activity [205].

**Table 2:** Effects of inhibitors on desalinization in the esophagus of teleosts

Species	Mucosal side						Serosal side		Reference
	Amiloride (ENaC)	DMA/EIPA (NHE)	DIDS/DNDS (AE, NBC)	Bumet/Furo (NKCC)	HCTZ (NCC)	DPC (ClC)	Ouabain (NKA)	DPC (ClC)	
Winter flounder	+	ND	ND	–	ND	ND	++	ND	[166]
Japanese eel	ND	ND	+	+	+	ND	++	ND	[157]
Gulf toadfish	–	+	ND	ND	–	ND	ND	ND	[49]
Japanese eel	ND	++	+	–	++	–	++	++	[227]

+, effective; −, ineffective. Number of + indicates strength of the effect. In parenthesis is target transporter of the inhibitor. For details, see text and abbreviation list. *Bumet* bumetanide, *Furo* furosemide, *ND* not determined

### 2.1 Molecular mechanisms of desalinization

**Fig. 2:**
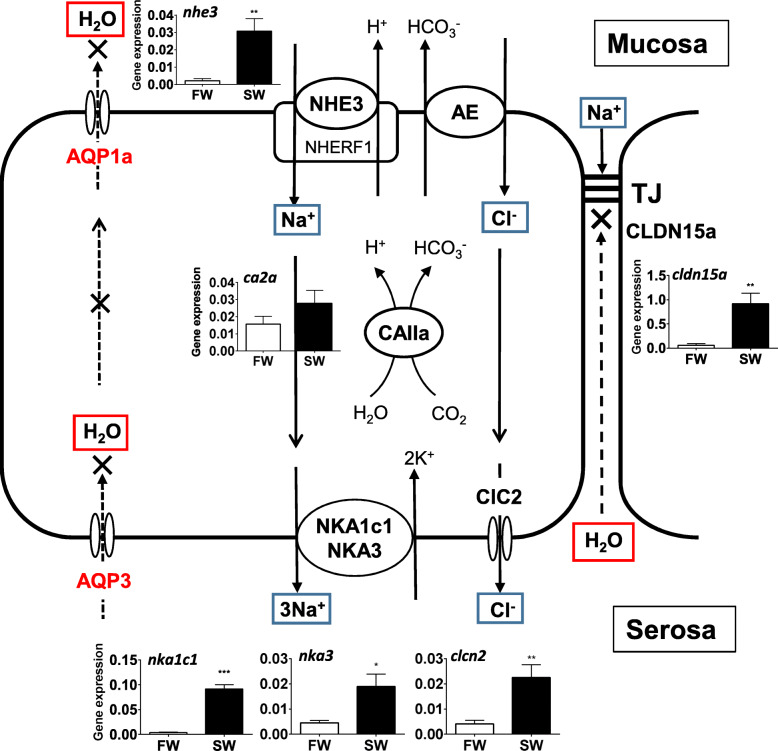
Major transporters responsible for desalinization (NaCl absorption) and low water permeability in the esophageal epithelia of SW eels. CA provides H^+^ and HCO_3_^−^ to facilitate coordinated action of NHE and AE for NaCl absorption. Upregulation of the genes is shown in the inserted figures. The molecular identity of AE is still unknown because of the lack of upregulated genes in SW. AQP genes are downregulated in SW. For details, see 2.1. For abbreviation definitions, see the list. Modified from Takei et al. [227]

Transporter molecules involved in desalinization have been assessed by transcriptome analysis (RNA-seq) using the esophagi of FW- and SW-acclimated eels [227]. Among the candidates implied by the inhibitor studies, the candidates were further narrowed down with criteria of (1) sufficient expression in the esophagus and (2) upregulation in SW-acclimated eels. Since RNA-seq can hardly distinguish the expressed genes at the isoform level, all paralogs were mined from the eel genome database, and real-time qPCR was performed using paralog-specific primers after transfer of eels from FW to SW [227]. The final candidate transport proteins that met the criteria are shown in Fig. 2 together with the changes in the transcript levels after SW acclimation.

#### *Mucosal side*

For transcellular NaCl absorption, the major route on the mucosal side is via coupled NHE3 (SLC9a3) and AE on the apical membranes of epithelial cells in exchange for H^+^ and HCO_3_^−^ excretion into the lumen (Fig. 2). Several AE genes of the SLC26 and SLC4 families are expressed in the esophagus, of which apically located SLC26a3a/b (also called downregulated in adenoma, DRA) and SLC26a6a (also called putative anion transporter 1, PAT1) are candidates, but their expression levels are low and not upregulated after SW acclimation. DIDS-sensitive SLC4a2a (AE2a) is another candidate, as its gene is abundantly expressed in the eel esophagus as in the mammalian intestine [244]. Both apical and basolateral localization of AE2 have been reported in the mammalian intestine [174]. NHE3 and SLC26a3/6 may be bound together to the scaffolding protein NHERF1 (NHE regulatory factor 1) via the PDZ-binding motif to form a metabolon (see 6.3). Their activities may be regulated by NHERF1 through phosphorylation and/or vesicle trafficking of the complex to the apical membrane (see 6.4). The NHERF1 gene (*slc9a3r1*) is expressed significantly in the eel esophagus [227]. As various second messengers (cAMP, Ca^2+^ and cGMP) regulate the activity of the metabolon, hormonal regulation of esophageal desalinization most likely occurs.

The activity of coupled NHE3 and AE should be enhanced by the production of H^+^ and HCO_3_^−^ from CO_2_ hydration by cytosolic CAII, which is expressed abundantly in the esophagus in SW eels [227]. CAII is also known to bind to NHERF and to form a metabolon for its regulation (see 6.3). Two CAII genes (*ca2a* and *ca2b*) are expressed in the eel esophagus, and *ca2a* is more abundant and upregulated after SW acclimation (Fig. 2). Thus, CAIIa plays a major role in the production of H^+^ and HCO_3_^−^ for activation of NHE3 and AE. This is in contrast to the eel intestine, where CAIIb is a major cytosolic CAII that produces HCO_3_^−^ for its secretion into the lumen and carbonate precipitation (see 4.4.1 and 4.4.2).

In the gulf toadfish, the NHE1 (SLC9a1), NHE2 (SLC9a2), NHE3, CAII, SLC26a6 and AE2 genes are expressed in the esophagus, but none of the genes are upregulated after transfer of the fish from SW to concentrated 60 ppt SW [49]. EIPA inhibits desalinization, and among EIPA-sensitive NHEs, NHE2 is likely responsible for Na^+^ uptake given the reduced expression of the NHE3 gene in concentrated SW. On the other hand, *nhe2* is constitutively expressed in the esophagus in both FW and SW eels. Thus, NHE2 may also function to take up Na^+^ irrespective of environmental salinity, while upregulated NHE3 is specifically involved in the enhanced Na^+^ uptake in the SW eel esophagus. Recruitment of NHE3-tagged vesicles to the apical membrane has been reported to occur in response to stimuli via intracellular messengers in mammals (see 6.4). The differences in the major transporters used for esophageal desalinization may reflect the differences in osmoregulatory mechanisms among euryhaline migratory eels and stenohaline, sedentary marine toadfish [224].

#### *Serosal side*

On the serosal side of epithelial cells, Na^+^ and Cl^−^ that enter the cells are extruded into the extracellular interstitial fluid by the coordinated action of ouabain-sensitive NKA (NKA1c1 and NKA3) and DPC-sensitive Cl^−^ channel 2 (ClC2) on the basolateral membrane (Fig. 2). NKA1c1 and NKA3 are catalytic α-subunits of NKA, and these genes (*atp1a1c1* and *atp1a3*) are profoundly upregulated after SW acclimation (Fig. 2). The NKA1c2 and NKA1c3 genes are also expressed significantly in the esophagus, but no upregulation occurs after SW acclimation [227]. Thus, these NKAs may play a maintenance role in Na^+^ absorption in the esophagus in both FW and SW eels. Eels have no NKA1a and NKA1b subunits like those found in salmonid gills [85], but the NKA1c subunit is present and diversified into 3 isoforms in eels [270]. The K^+^-Cl^−^ cotransporter 1 (KCC1, SLC12a4) gene is also expressed significantly in both the FW and SW eel esophagus; this gene may be responsible for Cl^−^ extrusion and recycling of K^+^ accumulated by NKA in the cell on the serosal side. It is also possible that AE2 is involved in Cl^−^ extrusion in exchange for HCO_3_^−^, as is suggested to occur in the toadfish [49], as both apical and basolateral localization has been reported for mammalian SLC4-type AEs [174].

### 2.2 Water transport

Another feature of the SW eel esophagus is low water permeability [83]. The low water permeability through the epithelial cells is accounted for by the low expression levels of the two aquaporin genes *aqp1a* and *aqp3*, which are further downregulated after SW acclimation (Fig. 2). AQP1 may be on the apical membranes of epithelial cells, and AQP3 may be on the basolateral membrane, as suggested by the localization of these AQPs in the intestine (see 4.2.1). The amount of *aqp1a* transcripts in the esophagus is 1/50 that in the intestine. On the other hand, *aqp1dup*, most likely *aqp1b*, is expressed weakly in the esophagus in European eels and upregulated after cortisol treatment [149]. Plasma cortisol concentrations increase transiently after SW transfer, and cortisol administration increases intestinal water absorption in eels [84].

### 2.3 Paracellular pathway

The TJ protein genes *cldn1*, *cldn3b*, *cldn5b*, *cldn7b*, *cldn11b*, *cldn11b, cldn12*, *cldn15* and *cldn23a* are expressed in the esophagus, as detected by RNA-seq, of which *cldn3b, cldn5b*, and *cldn15* are upregulated in SW-acclimated eels [227]. TJs are composed of proteins from the claudin (CLDN) family and of TJ-associated MARVEL proteins such as occludin and tricellulin, but CLDNs are the primary proteins that determine paracellular ion and water permeability [78]. Among the expressed CLDN genes, *cldn15a* is exceptionally highly expressed, and its expression is profoundly upregulated (> 80-fold) after SW acclimation, as detected by RNA-seq; this upregulation has been confirmed by qPCR (Fig. 2). As CLDN15 is known to act as a cation channel in mammals [121], it may serve not only as a barrier for water movement but also as a paracellular route for Na^+^ uptake (Fig. 2) if it is also Na^+^-permeable in teleosts (see 6.1). The same sets of CLDN genes are expressed in the esophagus and intestine except that *cldn3b* is additionally expressed in the esophagus. All CLDNs expressed in the esophagus other than CLDN15 are barrier-forming CLDNs [121], and the amounts of all transcripts are much greater (~ 10-fold) in the esophagus than in the intestine. Thus, these CLDNs form a barrier and inhibit paracellular water transport in the SW eel esophagus (Fig. 2). Paracellular tightness can also be confirmed by the much higher transepithelial resistance of the esophagus than of the intestine in SW eels.

## 3.1 Do the stomach and pyloric caeca play roles in SW acclimation?

There is a sphincter at the exit of the stomach that keeps SW for further dilution in the stomach for some time before it is sent out to the anterior intestine [261]. The resultant stomach expansion inhibits further drinking in the eel [82]. Measurement of the drinking rate in the eel by the esophageal fistula method with reintroduction of ingested water into the stomach [226] has revealed that SW eels do not drink constantly but rather drink in a rhythmic pattern, i.e., with repeated increases and decreases in intervals of 15–30 min. This indicates that the sphincter is relaxed after the interval, which relieves stomach expansion and restores vigorous drinking in SW.

In some species, such as salmonids and sparids, diverticula grow from the anterior intestine to form small blind-end tubes known as pyloric caeca. The number of pyloric caeca varies among species, ranging from 5 to 6 in sea bream to > 200 in salmonids. The function of pyloric caeca has attracted attention since Aristotle’s era, and it was shown that this tissue serves as an extension of the intestine to increase the surface area for nutrient absorption [25]. Vigorous water absorption has been demonstrated to occur in the pyloric caeca of the chinook salmon, *Oncorhynchus tshawytscha*, and the uptake capacity is sixfold higher than that of the intestine in SW-acclimated fish [250]. In the gilthead sea bream, *Sparus aurata*, isolated enterocytes from pyloric caeca possess higher NKA activity than those from the intestine [45]. High NKA activity has also been found in the pyloric caeca of chinook salmon, and cortisol treatment further augments NKA activity and the capacity for water absorption [248]. In addition to NKA, high expression of *aqp8b* has been found in the pyloric caeca of the Atlantic salmon, *Salmo salar*, supporting the role of water absorption from imbibed SW in this tissue [235]. Epithelial conductance is higher in the pyloric caeca than in the anterior intestine in the rainbow trout *Oncorhynchus mykiss* [75], suggesting that precipitation of divalent ions could occur not only in the intestine but also in the pyloric caeca (see 4.4). The pyloric caeca may perform both desalinization and water absorption, but further studies are required to clarify the osmoregulatory roles of this intriguing tissue.

## 4. The intestine is an essential organ for water acquisition

After desalinization in the esophagus and subsequent minor processing in the stomach, ingested SW becomes only slightly hypertonic to body fluids when it enters the intestine. The major task of the intestine for osmoregulation is to absorb water together with NaCl using a suite of transporters, of which Na^+^-K^+^-2Cl^−^ cotransporter 2 (NKCC2, SLC12a1) is unique to marine teleosts (see 4.1). As SW contains high concentrations of divalent ions (Table 1) and as these ions are hardly absorbed by the intestine [166, 207], water absorption results in increased divalent ion concentrations in the luminal fluid. As 74–85% of water is absorbed by the intestine in SW eels [207], the concentrations of Mg^2+^ and SO_4_^2−^ in the luminal fluid become 250 mM and 150 mM, respectively, if 80% of the water is absorbed from SW. As a result, the osmolality produced only by these ions almost equals that of plasma, although the osmotic coefficient of MgSO_4_ (0.58) is lower than that of NaCl (0.93). The high osmolality produced by the divalent ions certainly inhibits additional water absorption. To overcome this problem, the marine teleost intestine secretes HCO_3_^−^ into the lumen and decreases Ca^2+^ and Mg^2+^ concentrations via precipitation of these ions as carbonates (see 4.4). In addition, Mg^2+^ and SO_4_^2−^ appear to be absorbed meaningfully by the intestine, as discussed in 6.2.

### 4.1 NaCl absorption

**Fig. 3:**
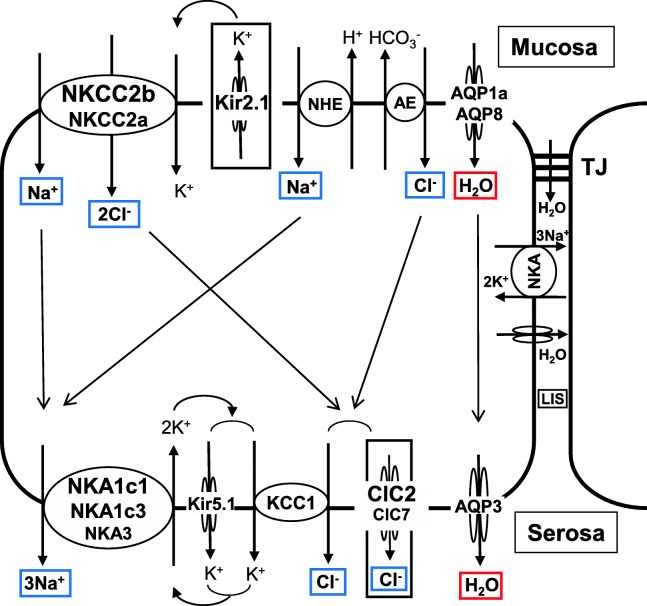
Major transporters responsible for Na^+^ and Cl^−^ absorption (blue rectangles) and water absorption (red rectangle) in the intestinal epithelia of SW eels. K^+^ extrusion at the mucosal side and Cl^−^ extrusion at the serosal side (black rectangles) produce the serosa-negative potential difference typical of the marine teleost intestine. For the molecular identities of NHE and AE, see Fig. 6. The local increase in osmolality in the lateral interspace (LIS) produced by NKA stimulates water flux into the space through the AQP and tight junction (TJ). The flows of ions and water within the cell are shown by arrows. The text size for the transporters indicates the relative abundance and upregulation of the transporters in SW. For details, see 4.1.1. For abbreviation definitions, see the list

Initial studies have shown that K^+^ in the luminal fluid is essential for NaCl absorption in the intestine in winter flounder [156] and SW-acclimated eels [6]. The stoichiometry of absorbed ions is 1Na^+^: 1 K^+^: 2Cl^−^ and thus electroneutral [164]. Furthermore, NaCl absorption is completely blocked by mucosal application of furosemide or bumetanide, each of which is an NKCC inhibitor, in the intestines of SW-acclimated eels [7, 12]; red drum, *Sciaenops ocellatus* [50]; and other marine teleosts (see [69, 133]). These results suggest that the major transporter on the apical membrane responsible for NaCl absorption is an NKCC, probably NKCC2. As NKCC2 takes up four ions (osmolytes) into the epithelial cell at a time, water efficiently moves in parallel into the cell through AQPs (see 4.2). Mucosal application of Ba^2+^, a K^+^ channel inhibitor, also blocks NaCl absorption so that recycling of K^+^ back into the lumen via a K^+^ channel is necessary for continuous functioning of NKCC2 [57]. A shortage of K^+^ in the lumen easily develops because of the low concentration of K^+^ in SW compared with the concentrations of Na^+^ and Cl^−^ (Table 1). K^+^ efflux at the mucosal side and Cl^−^ efflux at the serosal side produce a serosa-negative transepithelial potential difference (PD), which is characteristic of the marine teleost intestine (Fig. 3). In mammalian intestines, dominant Na^+^ influx at the mucosal side by epithelial Na^+^ channels (ENaCs) produces serosa-positive PD [106]. In the distal intestine, including the rectum, NCC, more accurately NCC1 (SLC12a3), is also involved in NaCl absorption in the eel intestine (see 4.1.1).

As observed in the esophagus, Cl^−^ is also taken up by AE in exchange for HCO_3_^−^[68, 69]. This mechanism is supported by the fact that removal of Cl^−^ from the mucosal fluid inhibits NaCl absorption (which also inhibits NKCC2) and the fact that AE is responsible for HCO_3_^−^ secretion into the lumen for carbonate precipitation (see 4.4). However, mucosal application of DIDS, an AE inhibitor, minimally inhibits NaCl absorption in the intestine in SW-acclimated eels [7] and the goby *Gillichthys mirabilis* [43]. In addition, more than 50% of Cl^−^ absorption is accounted for by AE function in the toadfish and some other marine teleosts [68, 73]. To complete NaCl uptake by AE, Na^+^ is taken up in exchange for H^+^ by NHE, as observed in the esophageal epithelium (Fig. 2) and in the intestines of mammals (see 4.1.1). Collectively, the major apical transporters responsible for NaCl absorption are NKCC2 in the intestines of SW eels and other marine (euryhaline) teleosts, while the combination of AE and NHE plays a significant role in some marine species. This variation in responsible transporters also illustrates the diversity of osmoregulatory mechanisms among teleost species [224].

On the serosal side of the intestinal epithelium, ouabain, an NKA inhibitor, has been found to significantly block NaCl absorption in all teleost species examined thus far (see [69, 133]). Thus, a low cytoplasmic Na^+^ concentration produced by NKA is essential for the activity of NKCC2, NCC1 and NHE and important not only for NaCl absorption but also for HCO_3_^−^ secretion [71]. NKA is an electrogenic pump that extrudes 3Na^+^ in exchange for 2 K^+^ across the basolateral membrane. As a result, the resting membrane potential of enterocytes becomes negative, and K^+^ ia maintained in the cytosol. The accumulated K^+^ is recycled into the extracellular space via a K^+^ channel for continuous functioning of NKA, as the K^+^ concentration in teleost plasma is in the low millimolar range (Fig. 1). Cl^−^ channels have been suggested to be present on the basolateral membrane for Cl^−^ efflux into the extracellular space [135] to complete transcellular NaCl transport with NKA. This Cl^−^ efflux is the cause of serosa-negative PD in the marine teleost intestine, as mentioned above. The presence of KCC on the basolateral membrane, which extruces Cl^-^ and K^+^ to the extracellular fluid at the same time, has also been suggested [208].

Na^+^ and Cl^−^ are also transported via the paracellular pathway of the intestinal epithelium, which will be discussed in detail in section 6.1. The intestinal epithelia of marine teleosts are leaky and exhibit low transepithelial resistance (Rt), and the Rt increases from the anterior to posterior direction and is highest in the rectum in the eel [12] and in other teleosts [133].

#### 4.1.1 Molecular mechanisms of NaCl absorption

##### Mucosal side

**Fig. 4:**
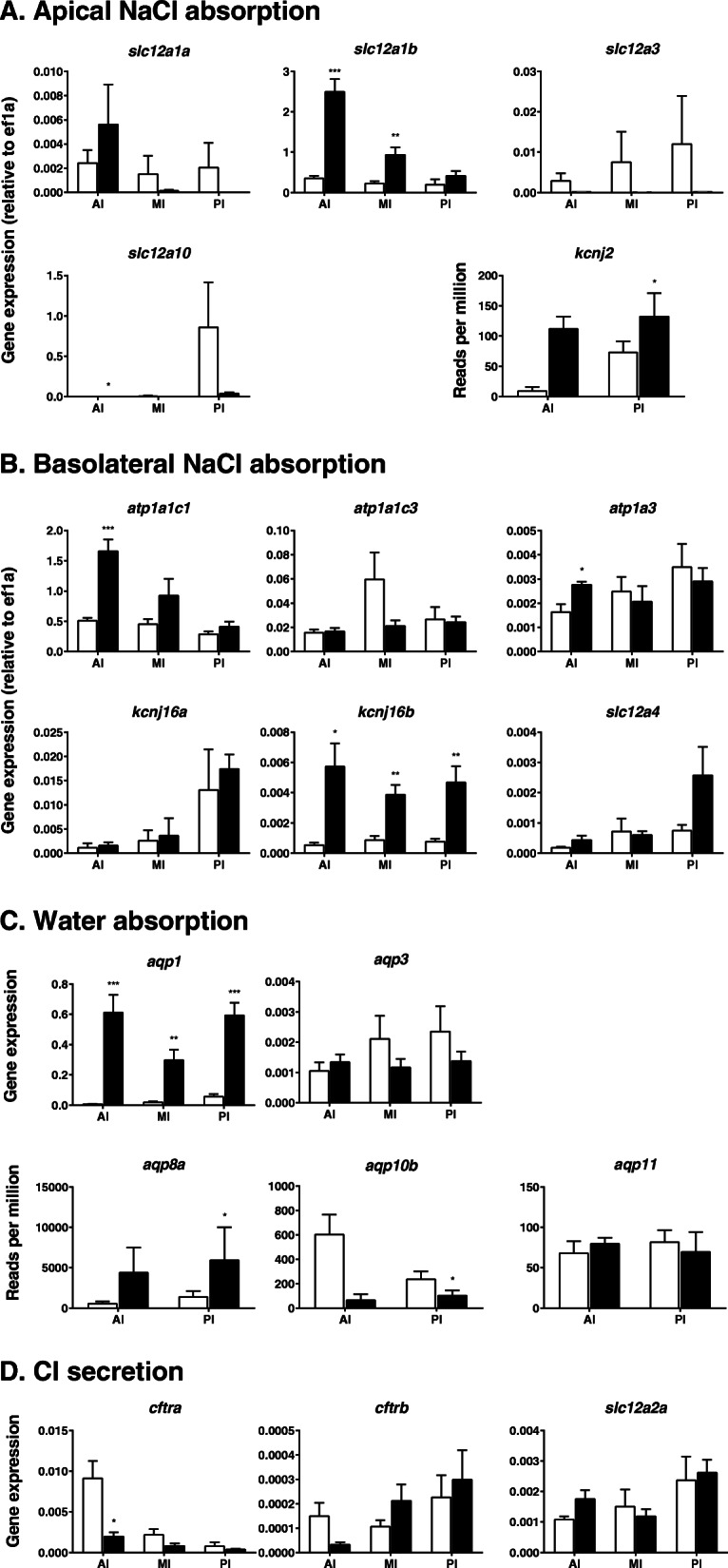
Expression of the genes responsible for NaCl (**A** and **B**) and water absorption (**C**) in the anterior intestine (AI), middle intestine (MI) and posterior intestine (PI) in FW-acclimated (plain column) and SW-acclimated (filled column) eels as determined by real-time qPCR. The expression of the genes related to Cl^−^ secretion suggested in mammals and other marine teleosts is also shown in (**D**). The expression levels are corrected by those of *elf1a* and thus indicate the relative abundance values of the genes. The *atp1*, *kcnj2* and *kcnj16* are the genes for NKA, Kir2.1 and Kir5.1, respectively. The figures with ‘Reads per million’ on the ordinate were created from RNA-seq data (n = 5). The figures are depicted based on the data in Ando et al. [12], Wong et al. [270], and Takei et al. [228] and on unpublished data. The primers for real-time PCR, including those for the unpublished data, are listed in Supplementary Table 1. *p < 0.05, **p < 0.01, ***p < 0.001. For details, see 4.1.1 and 4.2.1. For abbreviation definitions, see the list

Although only one NKCC2 exists in mammals, two isoforms, NKCC2a and NKCC2b, exist in the eel [37, 255], of which NKCC2b is the major isoform in the intestine (Fig. 3). Of the two NKCC2 genes, *slc12a1b* is expressed at a much higher level than *slc12a1a* and is upregulated in all intestinal segments in eels after acclimation to SW (Fig. 4): [12, 268]. The upregulation of *slc12a1b* occurs after transfer to SW or hypertonic medium in the intestines of all euryhaline and marine teleost species examined thus far, including the Mozambique tilapia, *Oreochromis mossambicus* [130]; olive flounder, *Paralichthys olivaceus* [111]; gilthead sea bream [66]; red drum [50]; and spotted sea bass, *Lateolabrax maculatus* [282]. The *slc12a1b* expression decreases gradually in the posterior direction in the intestine, while the NCC1 gene *slc12a3* expression increases gradually and is highest in the rectum in both European and Japanese eels [38, 255]. Thus, NCC1 may play a role in NaCl absorption in the rectum. However, *slc12a3* expression is lower in SW eels than in FW eels, and it is much lower than that of *slc12a1b* even in the posterior intestine (Fig. 4). The expression of *slc12a3* is also much lower than that of *slc12a1* in the intestines of Mozambique tilapia [130] and the three-spine stickleback, *Gasterosteus aculeatus* [140]. On the other hand, the NCC2 gene (*slc12a10*) is significantly expressed in the eel intestine, and the expression increases in the posterior direction (Fig. 4). NCC2 is involved in NaCl absorption in the gills of Mozambique tilapia and killifish [224] and probably in the NCC cells of the zebrafish, *Danio rerio* [90].

As mentioned above, active NKCC2 on the apical membrane causes K^+^ shortage in the luminal fluid, which inhibits continuous NKCC2 function. To compensate for K^+^ in the luminal fluid, K^+^ is recycled by a K^+^ channel, and Kir2.1 is the candidate (Fig. 3). The Kir2.1 gene (*kcnj2*) is expressed substantially in the eel intestine and upregulated after SW acclimation (Fig. 4). Na^+^ and Cl^−^ are also absorbed from the lumen into enterocytes via the coordinated action of AE and NHE. The molecular identities of AE and NHE are discussed in the HCO_3_^−^ secretion section (see 4.4).

##### Serosal side

Concerning basolateral transporters for NaCl absorption, NKA1c1 (*atp1a1c1*), NKA1c3 (*atp1a1c3*) and NKA1c3 (*atp1a3*) are responsible for Na^+^ absorption, as they are expressed at considerable levels in the eel intestine [270]. The *atp1a1c1* and *atp1a3* expression are upregulated in the anterior intestine after SW acclimation (Fig. 4). These NKAs may work together to extrude Na^+^ into the interstitial fluid in SW eels (Fig. 3).

To ensure the constant activity of NKA, K^+^ should be recycled into the extracellular fluid via a basolateral K^+^ channel, and Kir5.1 is the candidate (Fig. 3). Kir5.1 was first identified as a partner of NKA in the ionocytes of SW eel gills [216]. In fact, two Kir5.1 genes (*kcnj16a* and *kcnj16b*) are expressed in the eel intestine, and *kcnj16b* is upregulated in all intestinal segments of SW-acclimated eels (Fig. 4). In the small intestines of mammals, Kir7.1 is involved in recycling of K^+^ for continuous activity of NKA [167], while Kir5.1 and Kir4.1 are responsible for K^+^ recycling for NKA activity in the distal convoluted tubules of the kidneys [258].

Concerning Cl^−^ efflux on the serosal side, ClC2 and ClC7 appear to be responsible (Fig. 3) because their genes (*clcn2* and *clcn7*) are expressed in the eel intestine and upregulated in the anterior segment after SW acclimation (unpublished data). The *clcn2* expression is particularly substantial and profoundly upregulated in SW. The *clcn3* is also expressed in the intestine, but its expression is much lower than that of *clcn2* and does not increase after SW acclimation. In mammals, ClC2 is responsible for Cl^−^ efflux at the serosal side of the intestinal epithelium [170], but the cell polarity of localization changes depending on various factors (see 4.3.1). Notably, ClC2 is localized on the lateral membrane close to the TJ and regulates the paracellular permeability of ions and water in the intestinal epithelium in mammals [160]. In addition, the KCC1 gene (*slc12a4*) is expressed in the eel intestine, and its expression tends to increase after SW acclimation (Fig. 4). It seems likely, therefore, that KCC1 on the basolateral membrane is responsible for K^+^ recycling for NKA and Cl^−^ efflux into the interstitial fluid (Fig. 3). Notably, the suite of transporters for NaCl absorption develops in the intestine during smoltification in the Atlantic salmon, when the fish are still in FW but preparing for downstream migration to the sea [215].

##### Knowledge from mammals

In mammals, the major routes for NaCl absorption are (1) electroneutral absorption via combined activity of NHE and AE and (2) electrogenic absorption via ENaC at the mucosal side of the intestinal epithelium [61, 106, 199]. ENaC transports only Na^+^, which is the major source of serosa-positive PD in the mammalian intestine. The ortholog of ENaC is not identifiable in the teleost genome. Instead, a member of the acid-sensing ion channel (ASIC) family, a subfamily of the ENaC/degenerin superfamily, exists in teleosts. ASIC, which is localized on the apical membranes of ionocytes in FW trout gills, takes up Na^+^ from environmental FW in exchange for H^+^ through vacuolar-type H^+^-ATPase (VHA) [46]. The transcript of ASIC is not detectable in the intestines of eels even by transcriptome analysis (unpublished data).

Two NHE genes (*NHE2* and *NHE3*) are expressed in the mammalian intestine, of which *NHE3* is dominant, and *Nhe3*^−/−^ mice exhibit decreased NaCl absorption and mild diarrhea [192]. In addition, only NHE3, not NHE2, responds to intracellular messengers such as cAMP for recruitment of transporter-tagged vesicles to the apical membrane (see 6.4), which demonstrates a regulatory role of NHE3 [44]. The major AE genes expressed in the intestinal epithelium are *Slc26a3* and *Slc26a6. Slc26a3* is expressed throughout the whole length of the intestine, while the *Slc26a6* transcript is not detectable in the distal colon [106]. It has been suggested that SLC26a3 exchanges 2Cl^−^/1HCO_3_^−^ and that SLC26a6 exchanges 1Cl^−^/2HCO_3_^−^ in culture cells with transient expression, but the stoichiometry is still under debate [195]. SLC26a3 plays a major role in NaCl absorption, as *Slc26a3*^−/−^ mice suffer from severe chloride-losing diarrhea [193]. The effect of *Slc26a6* knockout on the intestine is not as strong as the effect on the kidneys [200]. NHE and AE form a metabolon with other transporters and enzymes, as discussed in 6.3.

The transporters involved in intestinal NaCl absorption in mammals are similar to those of some marine teleosts but different from those of other teleosts, including eels, in which NKCC2 plays a dominant role (Fig. 3). Interestingly, NKCC2-based NaCl absorption is similar to the absorption in the thick ascending limb of Henle’s loop (TAL) in the mammalian kidneys, in which NKCC2 and renal outer medullary potassium channel (ROMK) on the apical membrane and NKA, ClC-Kb and KCC4 on the basolateral membrane are involved in transcellular NaCl absorption [16]. The advantage of the NKCC2-based method in teleosts is the efficient transport of NaCl and water, as mentioned above, but NKCC2 has no regulatory role in acid-base balance, unlike the combination of AE and NHE. It is possible that teleosts acquired the NKCC2 system in the intestine during the evolution of their habitat toward the ocean; this is particularly applicable to migratory/euryhaline fishes, which must cope with abrupt changes in environmental salinity.

### 4.2 Water absorption

In parallel with NaCl transport, water moves from the intestinal lumen into the body fluid through the epithelium if the luminal fluid is almost isotonic to the body fluid [259]. The transcellular and paracellular routes are possible routes for water absorption. As the plasma membrane consists of a lipid bilayer that is almost impermeable to water, AQPs on the apical and basolateral membranes greatly facilitate transcellular water absorption [95]. The possibility of water cotransport by transporters such as KCC and the Na^+^-glucose cotransporter has been suggested [281], but this idea is still under debate. Regarding the paracellular route, it is known that some TJ proteins have channel-like activity [121], and CLDN2 has been suggested to serve as a water channel (see 6.1). However, paracellular water flux seems to be minor in the intestine of the killifish [274].

The marine teleost intestine can absorb water from slightly hypertonic luminal fluid [62, 68, 194], which is also true in SW-acclimated eels [206]. However, a high MgSO_4_ concentration in the luminal fluid certainly limits water absorption and survival in the hypersaline environment in the toadfish [62]. The luminal fluid close to the apical membrane exists in a microenvironment covered by mucus and thus may be made hypotonic to the body fluid by H_2_O formation and carbonate precipitation catalyzed by extracellular CAIV (see 4.4.2). On the basolateral side, NKA extrudes 3Na^+^ in exchange for 2 K^+^, which generates an osmotic gradient between the intracellular fluid and extracellular fluid. If this occurs in the lateral space between the adjacent enterocytes just below the TJ (called the lateral interspace, LIS), the fluid in the LIS becomes hypertonic to both the luminal and intracellular fluids. Then, water moves from both compartments into the interstitial fluid passively [42, 126]. Although ion fluxes precede that of water to produce a driving force, the fluid transported across the epithelium is isotonic, which generates the solute (Na^+^)-recirculation model [125]. It is now known that Na^+^ recirculation is achieved by NHE on the basolateral membrane, not NKCC1, in the eel (see 4.4.1). The mechanisms of water absorption across intestinal epithelia have been discussed in detail by Whittamore [259].

#### 4.2.1 Molecular mechanisms of transcellular water absorption

Transcellular water movement is achieved through AQPs. At least four AQPs are expressed in the eel intestine, AQP1, AQP3, AQP8, and AQP10 [13, 110, 149], of which AQP3 and AQP10 are glyceroaquaporins that allow passage not only of water but also of glycerol, urea, etc. [95]. Two isoforms usually exist for AQP1 (AQP1a and AQP1b) in the eel and other teleosts. The expression of *aqp1a* has been found to be upregulated in all segments of the eel intestine [13] and in vascular endothelial cells [149]. The tissue distribution of *aqp1b* transcripts has not yet been examined in eels. The *aqp1* expression is most abundant in the rectum, where water absorption may be enhanced by increased hydrostatic pressure [109]. A valve-like structure exists between the posterior intestine and the rectum in eels, which blocks backflow when the luminal pressure in the rectum increases. AQP1 is localized on the apical membranes of intestinal epithelial cells, as indicated by immunohistochemistry of the intestines of SW-acclimated Japanese and European eels [13, 149]. Our recent study using transcriptome analysis (RNA-seq) showed that *aqp1a, aqp3, aqp8a, aqp10b* and *aqp11* were expressed in the eel intestine, of which *aqp1a* and a*qp8a* were expressed at high levels (Fig. 4). Isoforms were identified by a reverse-BLAST best hit approach in our eel transcriptome data (DDBJ accession number: DRA004258) using annotations in the zebrafish database (https://zfin.org/). From the RNA-seq data, the expression of *aqp1a, aqp3* and *aqp8a* was significantly upregulated after SW acclimation, but real-time qPCR showed that *aqp3* expression did not change after SW acclimation (Fig. 4), suggesting the possible presence of isoforms. On the other hand, our qPCR analysis showed that *aqp1a* was expressed in all intestinal segments and profoundly upregulated after SW acclimation in the eel (Fig. 4).

In the Atlantic salmon [236] and gilthead sea bream [172], both *aqp1a* and *aqp1b* are expressed in the intestine, with *aqp1a* exhibiting higher expression than *aqp1b*, and the expression of these genes is higher in fish acclimated to SW than in those acclimated to FW. Immunoreactive AQP1a is localized on both the apical and lateral membranes of sea bream enterocytes, while it is found on the basolateral membrane in the Atlantic salmon. It has been suggested that the direction of trafficking of transporter-tagged vesicles to either the apical or basolateral membrane is dependent on environmental salinity (see 6.4). Upregulation of *aqp1* has also been reported in the intestine of the silver sea bream, * Sparus sarba* [40], and the European sea bass, *Dicentrarchus labrax* [64].

The *aqp3* is most abundantly expressed in the gills of teleost fishes, but significant expression has also been detected in the intestine of the eel [149], and the expression level is higher in the rectums of SW-acclimated fish than in those of FW fish [110]. The *aqp3* expression is low in the intestines of Mozambique tilapia [254] and sea bass [64]. Among AQPs expressed in the eel intestine, AQP3 is the only AQP that has been found to localize on the basolateral membranes of epithelial cells [39, 254], although AQP3 also localizes to the apical membrane in killifish [181]. In mammals, apical AQP2 and basolateral AQP3 are responsible for transcellular water absorption at the renal collecting duct [102].

AQP8 is another candidate apical membrane AQP (Fig. 3). The *aqp8* is expressed in the eel intestine, and the transcript levels increase after SW acclimation [110, 149]. We have also observed upregulation of *aqp8a* in the posterior intestines of SW eels (Fig. 4). Immunohistochemical analysis showed that AQP8 exists on the apical membranes of intestinal cells. Three AQP8 genes (*aqp8aa*, *aqp8ab* and *aqp8b*) are expressed in the intestine in Atlantic salmon, of which *aqp8ab* has the highest transcript levels in the intestine, and *aqp8ab* is upregulated after SW acclimation [236]. The salmon AQP8ab is also localized on the mucosal (apical) side in enterocytes and appears to play important roles in water absorption [47], as is the case for mammalian AQP8 [240].

*The aqp10* expression is observed in the intestines of eels and is upregulated in SW-acclimated fish [110, 149]. However, our transcriptome data suggest that *aqp10b* is downregulated in the SW eel intestine (Fig. 4). The *aqp10* expression has also been reported in the intestines of Atlantic salmon [236] and gilthead sea bream [185]. Cellular localization of AQP10 has not yet been examined in fish, but AQP10 is localized on the apical membranes of enterocytes in humans [154]. Expression of *aqp3, aqp8* and *aqp10,* including their subtypes, has also been reported in the intestine of the three-spine stickleback [140].

To summarize the possible transcellular pathway for water absorption, the major apical AQP may be AQP1a in the eel intestine, as judged by the expression level of its gene and upregulation after SW acclimation (Fig. 3). In other marine teleosts, AQP8 and AQP10 on the apical membrane are also involved in the uptake of water into epithelial cells. The water taken up into the cells may be transported into the extracellular fluid via AQP3 on the basolateral membrane, although AQP3 gene expression is low compared with that of other AQPs. It seems that AQP1 also plays a role in water absorption at the serosal side in the Atlantic salmon [236]. In the euryhaline medaka, *Oryzias latipes*, *aqp1a*, *aqp7*, *aqp8ab* and *aqp10a* are downregulated after SW transfer, and immunoreactive AQP1a and AQP10a move from the apical membrane to the subapical region after transfer to SW, indicating decreased transcellular water permeability across the intestinal epithelium [139]. Thus, it is hypothesized that water is transported mostly via the paracellular route after SW acclimation in medaka, although paracellular water permeability appears to be suppressed in salmonids after SW acclimation [213]. The water permeability of TJ proteins is discussed in 6.1.

### 4.3 Cl^−^ secretion

As shown in Table 1, SW contains similar concentrations of Na^+^ (450 mM) and Cl^−^ (524 mM). As esophageal desalinization removes Na^+^ and Cl^−^ equally from SW, Na^+^ and Cl^−^ concentrations in the luminal fluid may also be similar when ingested SW enters the intestine for absorption. In the anterior intestine, NKCC2 takes up 1Na^+^ and 2Cl^−^ from the luminal fluid, and AE further takes up Cl^−^ in exchange for HCO_3_^−^ (Fig. 3). Accordingly, the amount of Cl^−^ in the luminal fluid decreases much faster than that of Na^+^ during passage along the intestinal tract. However, the concentration of Cl^−^ in the luminal fluid is higher than the Na^+^ concentration in the intestine and rectum in eels (Table 1) and other marine teleosts [4, 69, 182]. This implies that Cl^−^ is secreted into the lumen to maintain the activity of NKCC2 and AE for constant NaCl absorption.

In mammals, Cl^−^ secretion has been demonstrated in crypt cells of the intestine (see [61]). The secretory-type cells were once thought to be restricted to the crypt region of the intestine, but it was later shown that they are present more widely along the crypt-villus axis [98]. Accordingly, it is controversial whether the same enterocytes have both absorptive and secretory functions or whether two different cell types exist. In the eel, guanylin has been shown to inhibit NKCC2 and stimulate apical Cl^−^ channels at the same time via a single second messenger, cGMP, resulting in Cl^−^ secretion [9]. Thus, a single enterocyte seems to be able to change its function from absorption to secretion (see 5). However, a small population of secretory-type enterocytes appear to exist in the killifish intestine [145].

**Fig. 5:**
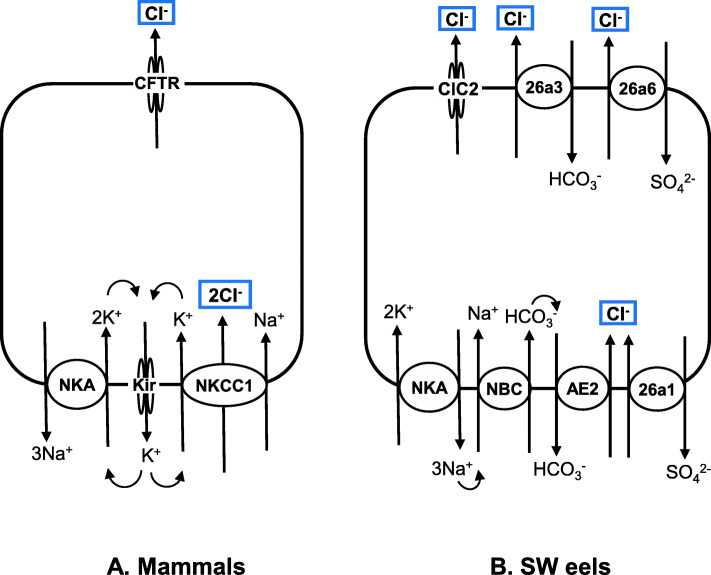
Transporters involved in transcellular Cl^−^ secretion (blue rectangles) into the lumen by intestinal epithelial cells in mammals (**A**) and seawater (SW)-acclimated eels (**B**). Because of the low expression of NKCC1 and CFTR in the intestines of SW eels, alternative molecular mechanisms are suggested. For details, see 4.3.1. For abbreviation definitions, see the list

Typical secretory-type cells are characterized by the presence of cystic fibrosis transmembrane regulator anion (Cl^−^) channels (CFTRs) on the apical membrane and NKCC1 (SLC12a3) on the basolateral membrane in mammals (Fig. 5). Low cytosolic Na^+^ caused by NKA promotes the activity of NKCC1 to take up Cl^−^ from the extracellular fluid into the cell. Then, increased cytosolic Cl^−^ is secreted into the intestinal lumen via CFTR, which is facilitated by the negative intracellular potential produced by NKA. Anion secretion accompanies parallel fluid secretion into the lumen, resulting in secretory diarrhea in mammals [61]. The suite of transporters for Cl^−^ secretion in the intestine is similar to those of mitochondrion-rich ionocytes of the gills in marine teleosts [85, 86] and secretory epithelial cells of the rectal gland in marine elasmobranchs [203]. In this sense, the rectal gland is similar to the crypt cells of the mammalian colon.

#### 4.3.1 Possible molecular mechanisms

In the teleost intestine, Marshall et al. [146] found substantial expression of *cftr* as high as that in the gills of SW-acclimated killifish. Apical localization of immunoreactive CFTR occurs only in the enterocytes of SW fish [145]. The enterocytes with CFTR immunoreactivity at the brush border membrane compose 20% of the total enterocyte population, suggesting the presence of secretory-type cells (Fig. 5). As immunoreactive CFTR migrates to the basolateral membrane in FW, the function of CFTR may change from Cl^−^ excretion in SW to Cl^−^ absorption in FW (see 6.4). Substantial expression of *cftr* has also been detected in the intestines of Mozambique tilapia [130] and spotted sea bass [282], and the expression is enhanced in hypersaline environments in these fishes. The *cftr* expression has also been reported in the intestines of gilthead sea bream [66], three-spine stickleback [140], and European sea bass [4]. In the sea bream, *cftr* expression is downregulated in the anterior intestine after transfer from diluted 12-ppt SW to regular SW or to hypertonic 55-ppt SW, but it is upregulated in the rectum.

The partner of apical CFTR for transcellular Cl^−^ secretion is basolateral NKCC1, as mentioned above. This coupling may also be the case in the intestines of some teleost species. The expression of two NKCC1 genes (*slc12a2a* and *slc12a2b*) has been demonstrated in the intestine of the spotted sea bass [282]. The expression of both NKCC1 genes in the intestine is much lower than that in other tissues and much lower than that of the CFTR gene. Furthermore, the expression of the major isoform, *slc12a2a*, does not change after acclimation to a hypersaline medium. Two NKCC1 genes are also expressed in the intestine of the gilthead sea bream [66], three-spine stickleback [140], and European sea bass [4]. Similar to *cftr* expression, *slc12a2* expression is downregulated in the anterior intestine of the sea bream but upregulated in the rectum after transfer from an isotonic to a hypertonic medium.

In contrast to the roles in these teleost species, the roles of CFTR and NKCC1 in Cl^−^ secretion seem to be minor in the eel. The expression of two *cftr* isoforms (*cftra* and *cftrb*) is detectable along the intestinal tract in Japanese eels [269]. However, the expression of *cftra*, a dominant isoform in the intestine, is low compared with that of *clcn2* and *clcn7*, which are upregulated in SW-acclimated eels (Fig. 4). Furthermore, the expression of *cfrta* is downregulated in the anterior intestine in SW-acclimated fish (Fig. 4). It is possible that CFTR of the FW eel intestine is localized in the basolateral membrane for Cl^−^ absorption, as suggested in killifish [145]. Gene expression does not always parallel protein abundance in cells, and it is possible that CFTR protein resides on the apical membrane or is stored in vesicles in the subapical region in enterocytes for recruitment after stimulation. In both FW and SW eel intestines, however, immunoreactive CFTR is not found on the apical membrane but rather in cytoplasmic vesicles [269]. Thus, it is likely that apical Cl^−^ channels different from CFTR may exist for Cl^−^ secretion in the eel. The presence of DPC-inhibitable and guanylin-sensitive Cl^−^ channels has been shown on the apical brush-border membrane of the eel intestine (see 5). ClC2 is usually localized to the basolateral membrane for Cl^−^ absorption in the intestines of mammals [28], as illustrated in Fig. 3. However, guanylin stimulates Cl^−^ secretion in the intestines of *Cftr*^−/−^ mice, where apical ClC2 is suggested to compensate for CFTR for Cl^−^ secretion (see [56]). As the polarity of vesicle recruitment changes depending on the environmental stimuli, as suggested for CFTR [145], it is possible that ClC2 is responsible for Cl^−^ secretion stimulated by guanylin (unpublished data). Alternatively, it is also possible that SLC26a6 on the apical membrane excretes Cl^−^ in exchange for SO_4_^2−^ driven by its high concentration (> 100 mM) in the luminal fluid (see 6.2) or that SLC26a3 excretes Cl^−^ in exchange for HCO_3_^−^ driven by its high concentration (~ 100 mM) in the luminal fluid (see 4.4.1). SLC26a6 in the SLC26 family has a high affinity for SO_4_^2−^ [199], and it actively exchanges SO_4_^2−^ and Cl^−^ in the renal proximal tubules of eels [256], while SLC26a3 readily exchanges Cl^−^ and HCO_3_^−^ [199].

Concerning NKCC1 on the basolateral membrane, only *slc12a2a* is expressed in the intestines of both European eels [37] and Japanese eels [271]. Compared with that of the NKCC2 gene (*slc12a1b*), *slc12a2a* expression is low, and it does not change after SW acclimation (Fig. 4). In addition, *slc12a2a* expression is higher in silver eels ready for downstream migration than in river-dwelling yellow eels [37]. Because of the low *slc12a2* expression in the eel intestine, sulfate anion transporter 1 (SAT1, SLC26a1) could be a candidate for Cl^−^ uptake at the serosal side in exchange for SO_4_^2−^, which is facilitated by uptake of SO_4_^2−^ from the luminal fluid by SLC26a6 (Fig. 5, see 6.4). Cl^−^/SO_4_^2−^ exchange activity has been demonstrated to occur in the basolateral membrane of the rabbit intestine [191].

When all the data obtained thus far are taken together, it seems that secretory-type enterocytes, which have CFTR on the apical membrane and NKCC1 on the basolateral membrane, may exist in the marine teleost intestine for Cl^−^ secretion into the lumen (Fig. 5). This Cl^−^ compensation in the luminal fluid allows constant activity of NKCC2 and AE/NHE to be maintained for NaCl absorption and thus water absorption. This mechanism enables marine teleosts to absorb > 80% of water from ingested SW and explains why the luminal fluid Cl^−^ concentration is higher than the Na^+^ concentration in the luminal fluid of marine teleost intestine. However, knowledge from the eel intestine suggests that an alternative set of transporters is responsible for Cl^−^ secretion, which requires further clarification. Intestine-specific knockdown of a transporter gene can be performed evaluate the genes responsible for SW acclimation. The use of membrane-permeable antisense oligonucleotides injected directly into the intestinal lumen enables intestine-specific gene knockdown.

### 4.4 HCO_3_^−^ secretion and carbonate precipitation

The high CO_2_ concentration and high pH of the rectal fluid in marine teleosts were recognized 90 years ago by Homer W. Smith [207]. It was shown later that the high pH is caused by active HCO_3_^−^ secretion into the lumen by epithelial cells (see [68, 69, 259]). Luminal fluid alkalization is more profound after fish are acclimated to hypertonic SW [264], suggesting a role of HCO_3_^−^ secretion in SW acclimation. As will be discussed in detail below (see 4.4.2), the secreted HCO_3_^−^ helps precipitate carbonates of divalent ions (Mg^2+^ and Ca^2+^), which are present in SW at concentrations higher than those in plasma (Table 1) and further concentrated by water absorption. Precipitate formation decreases luminal fluid osmolality and further enhances water absorption [73]. It is important to note that carbonate precipitate formation by marine teleost intestines explains 3–15% of the total oceanic carbon cycle and contributes to the amelioration of ocean acidification via fixation of CO_2_ in the ocean [265]. Because of its important role in global sustainability, HCO_3_^−^ secretion by the marine teleost intestine is one of the recent topics of interest in fish physiology [48, 67, 80, 87].

The involvement of apical AE in HCO_3_^−^ secretion has been suggested by in vitro experiments using Cl^−^-deficient Ringer solution or application of DIDS on the mucosal side of the intestinal epithelium. Consistently, mucosal application of DIDS inhibits HCO_3_^−^ secretion in the sanddab, *Citharichthys sordidus*, and the rainbow trout [74, 75]. However, DIDS is more potent in inhibiting HCO_3_^−^ secretion when applied to the serosal side than to the mucosal side; it is only slightly inhibitory when applied to the mucosal side in the eel [7] and other teleost species [43, 52]. Such species differences may be due to the differences in major transporters used among species [224] and the different sensitivities of AEs to DIDS among teleost species, as shown in mammals (see below). HCO_3_^−^ secretion is significantly inhibited when Cl^−^-deficient Ringer solution is on the mucosal side [73, 263], and the presence of AE on the apical membrane is now widely recognized [68]. Concerning inhibition by serosal DIDS, as removal of serosal Na^+^ profoundly decreases HCO_3_^−^ secretion in the eel [7], Na^+^-HCO_3_^−^ cotransporter (NBC), a member of the DIDS-sensitive SLC4 family of HCO_3_^−^ transporters [174], may be involved in HCO_3_^−^ uptake from the serosal side for secretion into the lumen.

Two sources of HCO_3_^−^ in the cell are conceivable for HCO_3_^−^ secretion into the lumen. One is HCO_3_^−^ taken up from the serosal side by NBC [231] as mentioned above, facilitated by NKA-induced low cytosolic Na^+^ [68, 69, 71]. The other is endogenous HCO_3_^−^ produced in the enterocyte by hydration of CO_2_ catalyzed by cytosolic CAII [230]. Because of the high metabolic activity of the cells, CO_2_ production in intestinal epithelial cells is high. In fact, both sources contribute to the increase in cytosolic HCO_3_^−^ in the teleost intestine, while their relative contributions differ considerably among species [69]. Since serosal DIDS and removal of HCO_3_^−^ in the serosal fluid profoundly decrease HCO_3_^−^ secretion in the goby [43], eel [7] and other marine teleosts [262], the contribution of serosal HCO_3_^−^ is significant in these species. On the other hand, almost all HCO_3_^−^ is supplied by CO_2_ hydration in SW-acclimated rainbow trout [75]. CA is one of the enzymes that has the fastest reaction rate and catalyzes the following reaction: CO_2_ + H_2_O ⇌ HCO_3_^−^ + H^+^. The forward reaction is much faster than the reverse reaction, and in high-CO_2_ environments such as intestinal epithelial cells. Thus, the presence of cytosolic CAII greatly enhances HCO_3_^−^ production. Under high-HCO_3_^−^ and high-pH conditions, such as in the intestinal lumen, however, the reverse reaction occurs, which is accelerated by membrane-bound CAIV (see below).

#### 4.4.1 Molecular mechanisms of HCO_3_^−^ secretion

The molecular mechanisms of HCO_3_^−^ secretion have been investigated for more than a decade in several teleost species, and ample data have been accumulated [66, 69, 122, 231, 259]. In this section, however, we will first describe the data obtained in eels for comparison with those obtained in preceding studies on other teleost species.

##### Mucosal side

**Fig. 6:**
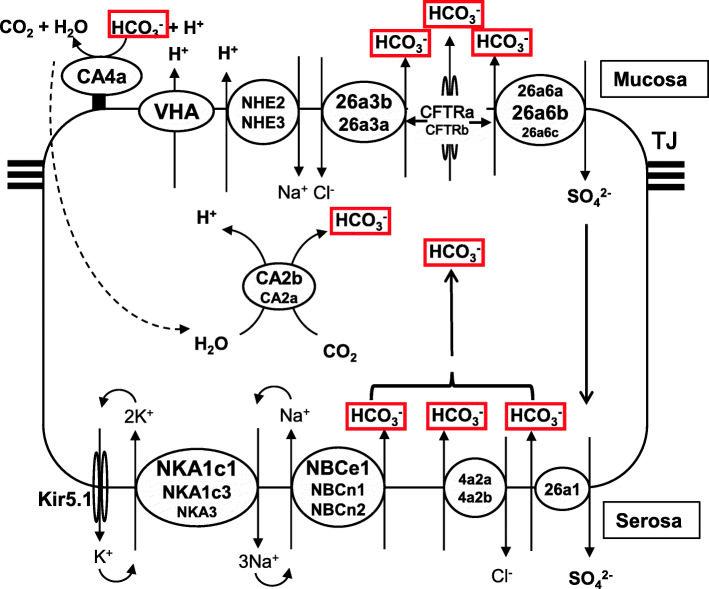
Major transporters responsible for HCO_3_^−^ secretion (red rectangles) into the lumen by the intestinal epithelial cells of SW eels. These transporters also function in NaCl absorption (see Fig. 3) and Mg/CaCO_3_ precipitation (see Fig. 8). NHEs, SLC26a3/6 s and CFTRs are combined as a metabolon for mutual activity regulation (see 6.3 and Fig. 8). The text size for the transporters is related to the relative abundance and upregulation of the transporters in SW. For details, see 4.4.1. For abbreviation definitions, see the list

**Fig. 7:**
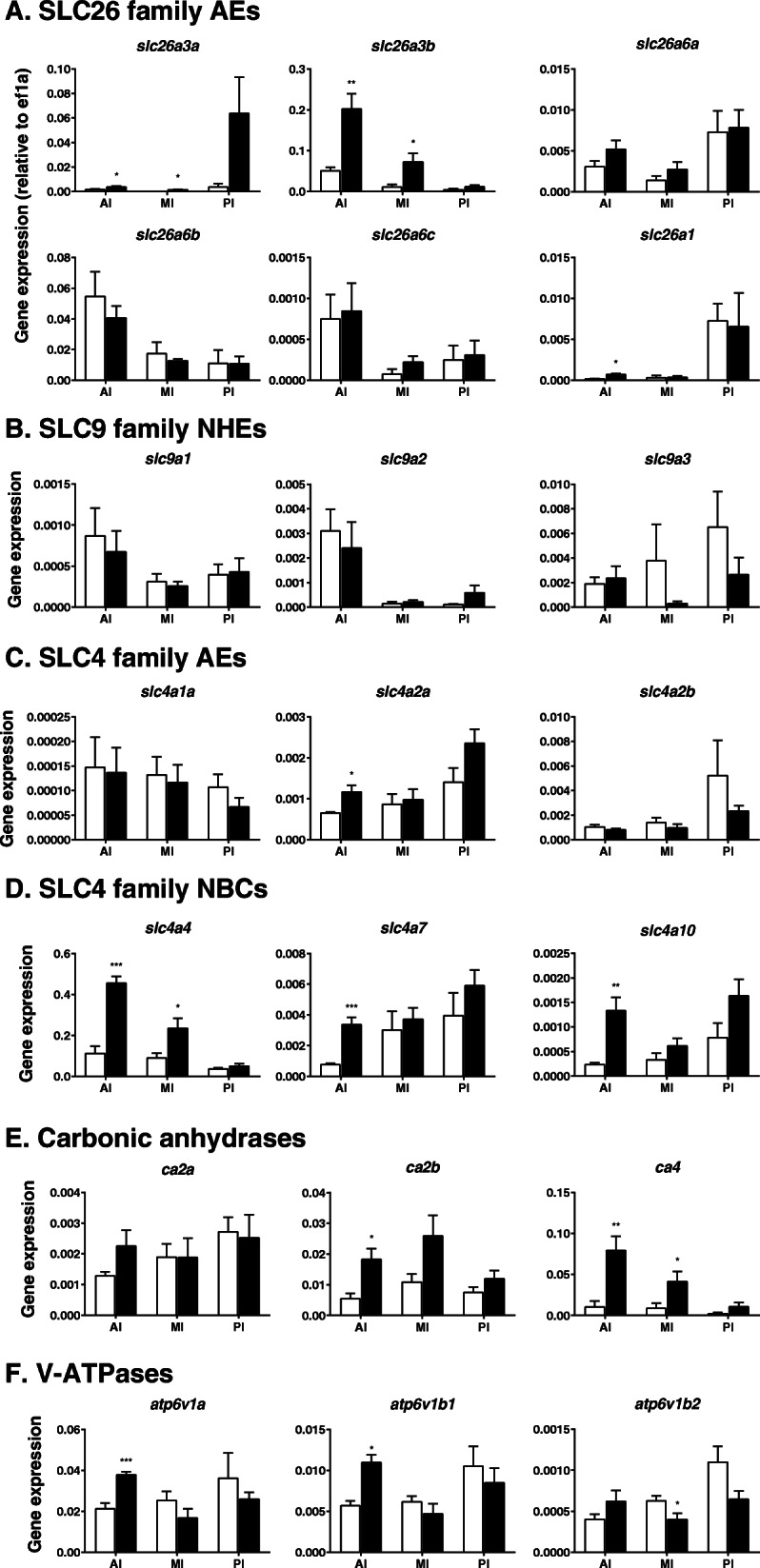
Expression of the genes responsible for HCO_3_^−^ secretion in the anterior (AI), middle (MI) and posterior (PI) intestine in FW-acclimated (plain column)  and SW-acclimated (filled column) eels as determined by real-time qPCR. The genes are grouped by SLC family and function. The *atp6*s are the genes for of VHA subunits. The figures are based on the data in Takei et al. [228] and Wong et al. [269] and on unpublished data. The primers for real-time qPCR, including those for the unpublished data, are listed in Supplementary Table 1. *p < 0.05, **p < 0.01, ***p < 0.001. For details, see 4.4.1. For abbreviation definitions, see the list

Three SLC26a6 genes (*slc26a6a*, *slc26a6b*, *slc26a6c*) are expressed at significant levels in the eel intestine, as assessed by transcriptomic analyses followed by real-time qPCR for differentiation of the isoforms (Figs. 6 and 7A). Among the isoforms, *slc6a6a* exhibits the most abundant transcripts in the posterior intestine, and the transcript levels decrease in the anterior direction; in contrast, *slc26a1b* transcripts are most abundant in the anterior intestine, and the transcript levels decrease in the posterior direction. The *slc26a6c* expression is the lowest among these isoforms in all intestinal segments. The *slc6a6a* expression is upregulated in all segments of the intestine during the course of SW acclimation [228], although the expression returns to the FW level after SW acclimation (Fig. 7A). The stoichiometry of SLC26a6 has been suggested to be electrogenic, exchanging 2HCO_3_^−^ with 1Cl^−^, in the mefugu [107], while variable stoichiometry has been reported in mammals [1, 106]. The electrogenic nature of SLC26a6 has also been shown in mefugu [122] and toadfish [76].

In addition, two SLC26a3 genes (*slc26a3a* and *slc26a3b*) are expressed abundantly in the eel intestine, and their expression is upregulated profoundly after SW transfer until the eels are fully acclimated to SW (Fig. 6 and 7A). The *slc26a3b* expression is most abundant in the anterior intestine and decreases in the posterior direction, while *slc26a3a* expression is most abundant in the posterior intestine and decreases in the anterior direction. In this way, two SLC26a3 isoforms and two SLC26a6 isoforms appear to compensate for each other in different segments of the intestine. The expression level of *slc26a3a/b* is several-fold higher than that of *slc26a6a/b* (Fig. 7A). The stoichiometry of SLC26a3 is reported to be HCO_3_^−^/2Cl^−^ in mammals (see [106]), but it has not yet been examined in teleosts. As mentioned above, however, mucosal DIDS fails to decrease HCO_3_^−^ secretion in the eel intestine [7, 228]. Although DIDS is generally an effective inhibitor of AE of the SLC26 family in mammals, it fails to block SLC26a3 in some species [15, 260], showing species specificity of DIDS for SLC26a3. Among teleosts, DIDS effectively blocks SLC26a6 in the rainbow trout [22], but its effect on SLC26a3 has not been confirmed in any teleost species. Supposing that DIDS is effective for SLC26a6 but ineffective for SLC26a3 in eels, the major apical AE of the SLC26 family responsible for HCO_3_^−^ secretion and Cl^−^ absorption might be SLC26a3 in eels.

**Fig. 8:**
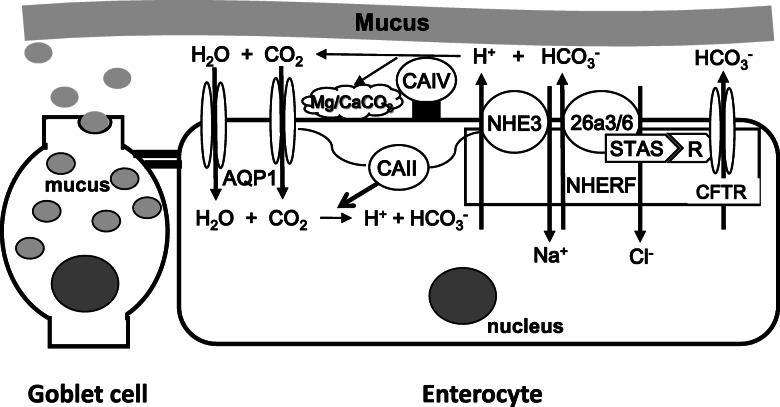
A suite of transporters and enzymes form a metabolon at the apical membrane of enterocytes for H^+^ and HCO_3_^−^ secretion and recycling to form carbonate precipitates in SW eels. VHA may also be involved in H^+^ secretion into the lumen (see Fig. 7). This idea of metabolon formation was first proposed in the mammalian intestine and may be applicable to the teleost intestine (see 6.3). H^+^ and HCO_3_^−^ secreted into the lumen by apical transporters are catalyzed by membrane-bound CAIV to produce CO_2_ and H_2_O, which are recycled into the cell via AQP1. Cytosolic CAII then produces H^+^ and HCO_3_^−^ again for continuous supply of the ions into the lumen. NHE3, SLC26a3/6 and CFTR are bound on NHERF for mutual regulation. CAIV also facilitates Mg/CaCO_3_ production in the microenvironment under the mucus layer, which is maintained by continuous secretion of mucus from goblet cells (see 4.5). CAII is known to bind to the NHE/AE complex and to AQP to regulate their activity. CFTR and SLC26a3/6 bind through the R domain and the STAS domain of each transporter. For details, see 6.3. For abbreviation definitions, see the list

Other candidate transporters for HCO_3_^−^ secretion are anion channels on the apical membrane, and *cftra* expression has been detected in the eel intestine (Fig. 4). CFTR is known to readily pass HCO_3_^−^ in mammals [204] and couple with SLC26a3/6 to stimulate HCO_3_^−^ secretion [212] (Fig. 8). The polarity (apical or basolateral) of CFTR localization seems to be variable depending on the environmental salinity and other conditions [145]. *clcn2* and *clcn3* are expressed significantly in the eel intestine (unpublished data). Significant expression of *clcn3* has also been reported in the cod intestine [87]. However, the major role of apical ClCs appears to be for Cl^−^ secretion in teleost fishes (see 4.3.1).

The involvement of SLC26a6 was first suggested in the euryhaline mefugu, in which *slc26a6* expression is profoundly upregulated after transfer from FW to SW [122]. Similar results have been reported in the rectum in gulf toadfish [179] and gilthead sea bream [66] transferred from diluted SW to concentrated SW. In contrast, *slc26a6* expression in the anterior intestine of Mozambique tilapia is suppressed after transfer from FW to SW [183]. In addition, *slc26a3* expression has also been detected in the intestines of several teleost species, and upregulation of the transcripts has been found in sea bream [66] and tilapia [183] after hyperosmotic stimulation. The s*lc26a3* and *slc26a6* expression has also been detected in the intestine of the European sea bass [4]. It seems that the relative contributions of SLC26a3 and SLC26a6 to HCO_3_^−^ secretion differ among teleost species.

As partners of SLC26a3 and SLC26a6 for coupled uptake of Na^+^ and Cl^−^ and excretion of H^+^ and HCO_3_^−^, three NHE genes, NHE1 (*slc9a1*), NHE2 (*slc9a2*) and NHE3 (*slc9a3*), are expressed in the eel intestine (Fig. 6 and 7B). NHE1 and NHE2 are resident-type, while NHE3 is a mobile-type transporter that is stored in cytoplasmic vesicles and inserted into the plasma membrane (see 6.4). The expression of NHE genes does not change in the eel intestine after SW transfer (Fig. 7B). AE and NHE are thought to be physically associated in the anchor protein (see 6.3). The gene of the anchor protein for NHE3, NHERF1 (*slc9a3r1*), is expressed significantly in the intestine, and its expression is upregulated twofold after SW acclimation, as demonstrated by RNA-seq (unpublished data). NHE3 and AE in the anchor protein are recruited from the cytoplasmic vesicles to the apical membrane upon phosphorylation (see 6.4). NHE3 is an electroneutral exchanger of 1Na^+^/1H^+^. With regard to other teleosts, *nhe3* expression has been detected in the intestines of rainbow trout [72] and cod [87].

##### Serosal side

On the basolateral membrane, SLC26a1 may be a major AE for HCO_3_^−^ uptake into the cell, as its gene expression is consistently upregulated in all segments of the eel intestine after SW transfer [228], although the upregulation is not significant in the middle and posterior intestine after full acclimation to SW (Fig. 7A). As SLC26a1 exhibits high affinity for SO_4_^2−^, it is possible that apical SLC26a6 and basolateral SLC26a1 work in concert for transcellular secretion of HCO_3_^−^ in exchange for SO_4_^2−^ absorption in the SW eel intestine (see 6.2). Cooperation of the two sulfate transporters has been observed in the proximal tubules of SW eel kidneys [257]. The SLC4 family of AEs also contains candidates for HCO_3_^−^ secretion: two AE2 genes (*slc4a2a* and *slc4a2b*) are expressed in the eel intestine, and *slc4a2a* tends to be upregulated after SW transfer (Fig. 7C). AE2 may be localized on the basolateral membrane, as assumed from its location in the intestines of mammals [174] and by the lack of inhibition of HCO_3_^−^ secretion by apical application of DIDS in the eel intestinal epithelium [228].

Among other HCO_3_^−^ transporters, three NBC genes, NBCe1a (*slc4a4a*), NBCn1 (*slc4a7*) and NBCn2 (*slc4a10a*), are expressed in the SW eel intestine, of which *slc4a4a* is expressed most profoundly (Fig. 7D). All the NBC genes are upregulated in different segments of the eel intestine after SW transfer [228]. Thus, HCO_3_^−^ may be taken up with Na^+^ from the extracellular fluid by electrogenic NBCela and electroneutral NBCn1 and NBCn2a. The stoichiometry of NBCe1 is 1Na^+^/2HCO_3_^−^ in mammals [174] and electrogenic in toadfish [231]. However, the external Na^+^ concentration affects the stoichiometry of mefugu NBCe1, and the ratio becomes 1Na^+^/4HCO_3_^−^ at 120 mM Na^+^, which is similar to the concentration in the extracellular fluid of teleosts [29]. NBCe1 activity is facilitated by the low levels of cytosolic Na^+^ produced by NKAs on the basolateral membranes of enterocytes [69, 133]. The *slc4a4* is also expressed in the intestines of mefugu [122], toadfish [231], and sea bream [66], and its expression is upregulated after transfer to hyperosmotic environments. The inhibition of HCO_3_^−^ secretion by serosal DIDS coincides with the DIDS sensitivity of NBCs [7, 43, 69]. Given all the results obtained thus far, it seems that transcellular HCO_3_^−^ transport occurs principally via apical SLC26a3 and SLC26a6 and basolateral NBCe1 and SLC26a1/AE2 in marine teleosts (Fig. 6).

##### Carbonic anhydrases and proton ATPase

In addition to uptake from the serosal side, cytosolic HCO_3_^−^ is provided by hydration of CO_2_ in epithelial cells, which is catalyzed by cytosolic CAII (CAc), as suggested in several teleost species [66, 75, 187]. CO_2_ is produced by active metabolism of enterocytes and is taken up from the luminal fluid (Fig. 8). Two cytosolic CAII genes (*ca2a* and *ca2b*) and a membrane-bound CAIV gene (*ca4a*) are expressed at high levels in the eel intestine, and *ca2b* and *ca4a* expression is upregulated after SW transfer (Fig. 7E). CAIV has a catalytic site on the luminal side and may produce H_2_O and CO_2_ from H^+^ and HCO_3_^−^ secreted into the microenvironment between the mucus layers and the apical membranes of epithelial cells (Fig. 8), as observed in rainbow trout [72]. H_2_O and CO_2_ production decrease luminal fluid osmolality to facilitate water absorption, and the generated CO_2_ is recycled into the cell for hydration by CAII (see 4.4.2). It is likely that CO_2_ is recycled efficiently via AQP1 on the apical membrane, as suggested in zebrafish [31] and mammals [171]. As HCO_3_^−^ secretion is inhibited to different degrees by mucosal application of acetazolamide, which blocks both CAII and CAIV at the same time, the relative contributions of the CAs to HCO_3_^−^ secretion differ among teleost species [69, 259]. The role of CAII in HCO_3_^−^ secretion has been documented in the rainbow trout [72] and other marine teleosts [69].

HCO_3_^−^ secretion is mediated by mutual activation of SLC26-family AEs and CFTR on the apical membrane (see 6.3). As SLC26a6 exchanges 1Cl^−^/n HCO_3_^−^ in teleosts [107], additional H^+^ secretion into the lumen neutralizes the secreted HCO_3_^−^ to support the activity of coupled SLC26a6 and NHE3, which exchanges 1Na^+^/1H^+^ [69, 76]. In fact, H^+^ secretion facilitates HCO_3_^−^ secretion and increases the production of CO_2_ and H_2_O by CAIV (see below). H^+^ secretion also helps protect against cellular acidification [76]. The candidate H^+^ transporter is VHA, which is expressed in the intestines of various teleost species, such as rainbow trout [72], toadfish [76], sea bream [66], cod [87], tilapia [183], Senegalese sole [182], and sea bass [4]. VHA is a large protein complex consisting of two domains, V0 and V1, that consist of six and eight subunits, respectively. Significant expression of the A subunit and 2B subunits in the V1 domain (*atp6v1a, atp6v1b1, atp6v1b2*) has been detected in the eel intestine. Among these subunits, *atp6v1a* and *atp6v1b1* are highly expressed, and their expression is upregulated after SW transfer (Fig. 7F). VHA also appears to be localized on the basolateral membrane to help maintain the resting potential and neutral pH of the cell [69]. The CAs and VHA appear to be physically coupled to the AE/NHE complex to form a metabolon (see 6.3).

#### 4.4.2 Mechanisms of carbonate production

Formation of white precipitates consisting of Ca/Mg carbonate within the intestinal lumen has been reported in various marine teleosts for more than 50 years; for example, such precipitates have been observed in southern flounder [81], rainbow trout [201], eels [88, 153, 207], toadfish [253], European flounder [262] and mefugu [122]. The solubilities of MgCO_3_ and CaCO_3_ in water are 1.65 and 0.13 mmol/l at 25 °C, respectively, while luminal fluid Mg^2+^ and Ca^2+^ concentrations are 188 and 14 mmol/l, respectively, in the eel intestine (Table 1). Although solubility changes greatly according to pH and the presence of other ions, concentrated HCO_3_^−^ in the luminal fluid certainly promotes the formation of carbonates [70, 265]. The chemical compositions of carbonate precipitates have been examined in teleost intestines. The carbonate precipitate consists of Ca carbonate and/or calcite in the flounder [81] and eel [88], and the calcite contains one-third (by weight) Mg and a small amount of Mn, producing a substance called calcian kutnohorite, in the gulf toadfish [253]. On the other hand, the carbonate precipitate contains 22.6 mol% Mg, 76.9 mol% Ca and small amounts of P and S and is called magnesian calcite (Mg calcite) in the eel [153]. Chemical and structural analyses have been performed on the crystalline products in the intestinal lumens of various tropical fishes and have revealed that Mg calcite contains 0.5 to more than 40 mol% MgCO_3_ [184]. The precipitate of the sea bream intestine contains 54 mol% Mg^2+^, and Mg^2+^ may be responsible for stabilizing the unstable mineral crystals [54]. The mechanisms of Mg calcite formation have been investigated in marine organisms, and high-Mg calcite that contains > 4 mol% MgCO_3_ is naturally formed by calcite-coated animals such as foraminifera, echinoids and corals [131]. The ratio of MgCO_3_ increases in proportion to the relative Mg^2+^ concentration in the medium. Recently, a protein-based matrix with strong Ca^2+^-binding properties was found in the teleost intestine; this matrix facilitates carbonate precipitation [188]. The proteins were purified from the Ca precipitates in the toadfish intestine and analyzed by proteomics. Many matrix proteins were found to be highly acidic, which may provide negatively charged domains for immobilization of Ca^2+^. Immobilization increases the local Ca^2+^ concentration in the microenvironment and facilitates the precipitation of divalent ions with secreted HCO_3_^−^.

As mentioned above, the space between the mucus layer and the apical membrane of enterocytes is a microenvironment different from the central lumen, which is maintained by constant secretion of mucus by goblet cells (Fig. 8). H^+^ and HCO_3_^−^ are secreted into the microenvironment, where not only CO_2_ and H_2_O production but also carbonate production are efficiently facilitated by membrane-bound CAIV via the following reaction: Ca^2+^/Mg^2+^ + 2HCO_3_^−^ ⇌ Ca/MgCO_3_ + H_2_O + CO_2_ [89]. This reaction not only removes divalent ions and produces H_2_O to decrease osmolality but also supplies CO_2_ through AQP1 for hydration to produce HCO_3_^−^ via cytosolic CAII for constant operation of apical AE (Fig. 8).

## 5. Hormonal control

The effects of hormones are governed by the presence of receptors in the tissue. Thus, it is important to examine the presence of hormone receptors in the digestive tract using a sensitive detection method such as RNA-seq. It is known that the gut is one of the organs that produces the greatest variety of hormones, and it synthesizes unique hormones such as guanylin in addition to those produced by other tissues such as brain-gut peptides [151, 222]. Considering the data obtained thus far, it is apparent that hormones that increase cAMP or cGMP as a second messenger inhibit NaCl and water absorption [8].

### 5.1 Hormones that inhibit NaCl transport

The most potent hormone that inhibits NaCl absorption is the cardiac hormone atrial natriuretic peptide (ANP), which almost nullifies short-circuit current (*Isc*) in the isolated intestinal epithelium of the eel when it is applied to the serosal side [10] and in the intestine of winter flounder [163]. The minimum effective dose of homologous eel ANP for inhibition of *Isc* is as low as 10^− 11^ M in vitro, which is much lower than those of other inhibitory hormones examined thus far [11] and much lower than the reported effective doses of ANP in the mammalian intestine [219]. The natriuretic peptide (NP) family is highly diversified in teleost fishes, and at least 7 members, ANP, B-type NP (BNP), ventricular NP (VNP), and 4 types of C-type NPs (CNP1 ~ 4), exist in the eel [93, 94]. Biological NP receptors (GC-A and GC-B) are single-stranded receptors with a cytosolic guanylyl cyclase (GC) domain that produces cGMP as an intracellular second messenger consequent to ligand binding [14]. Similar to the ligands, NP receptors are diversified in fishes compared with mammals [222]. The increased cGMP after ligand binding activates protein kinase GII (PKGII), which inhibits NKCC2 and decreases *Isc* by inhibiting K^+^ efflux on the mucosal side and Cl^−^ efflux on the serosal side (see Fig. 3), resulting in inhibition of NaCl absorption [10, 163]. As ANP and VNP are synthesized locally in the intestine, these hormones may act in a paracrine fashion as well as in an endocrine fashion [137].

Other hormones that potently inhibit NaCl absorption are the guanylin-family peptides, which are highly expressed locally in the intestine. The family members (guanylin, uroguanylin and renoguanylin) and their receptors (GC-C1 and GC-C2) are more diversified in the eel [34, 35, 278, 279] than in mammals, in which they have two ligands and one receptor. The guanylin family and its receptors form the only known hormonal system whose genes are all upregulated after transfer to SW. GC-C is another type of single-stranded membrane receptor with intracellular GC domain in addition to the NP receptors GC-A and GC-B. Homologous eel guanylin profoundly decreases *Isc* or even reverses it when applied to the mucosal side of the eel intestine [12, 277]. The *Isc* inhibition is due to inhibition of NKCC2, as is the case for ANP. Similar effects have been observed in the toadfish using eel renoguanylin, a heterologous hormone that is similar to toadfish guanylin [177]. The minimum effective dose for inhibition is 10^− 9^ M [12], which is higher than that for ANP [11]. This may be due to the paracrine action of guanylin from the lumen, into which it is secretedwith mucus from goblet cells and where it acts on GC-C localized on the apical membranes of enterocytes (acting in a luminocrine manner) [278]. Guanylin is more effective on the mucosal side than on the serosal side of the intestinal epithelium in vitro. Thus, guanylin is a local hormone in the intestine, although it is also secreted into the circulation. Guanylin and uroguanylin secreted into the circulation may act directly on the kidneys to inhibit NKCC2 at the TAL and induce diuresis/natriuresis [56].

More recent studies have shown that guanylin not only inhibits NKCC2 but also stimulates Cl^−^ channels on the apical membrane for Cl^−^ secretion into the lumen in the eel [9] and toadfish [177], resulting in reversal of *Isc*. In the eel, this effect is only detectable when fish Ringer solution on the mucosal side is replaced with a simulated luminal fluid in vivo that contains low NaCl and high MgSO_4_ and when NKCC2 activity is inhibited. The enhanced Cl^−^ secretion by guanylin occurs via DPC-sensitive Cl^−^ channels, not CFTR, in the apical membrane. In the toadfish, on the other hand, guanylin-induced Cl^−^ secretion appears to be mediated by apical CFTR and basolateral NKCC1, as in mammals [177, 180]. In the mammalian intestine, where NKCC2 is scarcely present on the apical membrane, guanylin induces Cl^−^ and HCO_3_^−^ secretion into the lumen through activation of apical CFTR [14, 56]. Guanylin inhibits Na^+^ absorption in mammals through inhibition of NHE3 and the scaffolding protein NHERF2, which are the major players in Na^+^ absorption in mammals (see 4.1.1). In toadfish, where AE and NHE play important roles in NaCl absorption, renoguanylin inhibits AE (SLC26a6) to decrease Cl^−^ absorption and HCO_3_^−^ secretion [178]. In contrast, guanylin stimulates HCO_3_^−^ secretion in the SW eel intestine, not through direct action on apical AE but through inhibition of NKCC2 [228]. NKCC2 inhibition decreases cytosolic Na^+^ and Cl^−^, which stimulate apical AE and/or basolateral NBC, resulting in increased HCO_3_^−^ secretion. Thus, guanylin supplies Cl^−^ to the lumen for continuous NKCC2 activity, increases HCO_3_^−^ secretion for carbonate precipitation, and flushes out carbonate precipitates from the rectum [12, 177], all of which facilitate SW acclimation, although inhibition of NKCC2 is certainly disadvantageous for SW acclimation. As reported recently, transfer of rainbow trout to SW stimulates intestinal motility [24]. As guanylin is upregulated after SW transfer in the eel and toadfish intestine and activates intestinal motility in mammals [55], the increased motility could be due to guanylin.

It has been suggested that guanylin-induced *Isc* inhibition is mediated not by cGMP but by cAMP in mammals [14]. This is because the cGMP increased by guanylin inhibits cAMP-specific phosphodiesterase 3 (PDE3), resulting in increased cAMP levels in enterocytes. Transcriptome analysis has shown that the PDE3 gene is also expressed in the eel intestine (unpublished data). Notably, the effect of renoguanylin on intestinal Cl^−^ and HCO_3_^−^ secretion is suggested to be mediated by PKA in the toadfish [180]. Thus, hormones that increase cytosolic cAMP inhibit *Isc* and therefore NaCl/water absorption in teleost fishes. This indicates that hormones that bind to G protein-coupled receptors (GPCRs) associated with the Gαs subunit are inhibitory to transport. Such hormones include vasoactive intestinal peptide, whose inhibitory action has been demonstrated in the intestines of winter flounder [162], Mozambique tilapia [143] and eels [11]. Urotensin I is also inhibitory in the goby [136], in which it binds to Gαs-associated GPCRs.

On the other hand, stimulation of soluble adenylyl cyclase (sAC) is suggested to be stimulatory for *Isc* in the toadfish intestine [237]. It has been shown that increased HCO_3_^−^ concentrations in Ringer solution on both sides of an Ussing chamber increase *Isc* and that this increase disappears following removal of mucosal Cl^−^ and mucosal application of bumetanide, suggesting an involvement of NKCC2. The findings also suggest that NKCC2 activation is induced by sAC because sAC inhibition abolishes the stimulatory effect of HCO_3_^−^ on *Isc* [237]. This idea is interesting and suggests a new role for sAC in the regulation of intestinal function. However, all hormones that increase cytosolic cAMP inhibit *Isc*. Calvalho et al. investigated this issue and found that transmembrane AC activated by hormones and sAC exert opposite effects on *Isc* in the sea bream intestine, although both ACs produce the same effector, cAMP, in the cytoplasm [26]. As mentioned above, NKCC2 governs the whole transport system in the intestines of most marine teleosts, and inhibition of NKCC2 profoundly affects other transporters and electrical parameters involved in intestinal transport, as exemplified by guanylin-induced Cl^−^ secretion [9] and HCO_3_^−^ secretion [228]. More data may be required to define the stimulatory effect of sAC on NKCC2, including data on the specificity of sAC inhibitors in teleosts.

### 5.2 Hormones that stimulate NaCl transport

In contrast to inhibitory hormones, hormones that decrease cAMP appear to increase *Isc* and NaCl absorption in the teleost intestine. Examples are somatostatin [245] and neuropeptide Y [246] in eels, whose receptors are GPCRs associated with the Gαi/o subunit that decrease cytosolic cAMP after ligand binding. Urotensin II and AVT also increase *Isc* in the intestines of tilapia [143] and sea bream [150]. These hormones bind to GPCRs associated with the Gαq/11 subunit and increase the levels of the second messengers IP_3_/Ca^2+^ and diacylglycerol (DAG). As the seabream intestine used in the above experiment shows low serosa-negative or serosa-positive PD, unlike the high serosa-negative PD in other marine teleosts [133], NKCC2 may be inhibited before AVT stimulation in the seabream intestine. For instance, the *Isc* of the intestine in SW-acclimated eels is ~ 500 μA/cm^2^ [12], while it is less than 5 μA/cm^2^ in the seabream intestine [150]. AVT does not affect *Isc* in the intestines of tilapia [142] or eels (unpublished data), which exhibit high serosa-negative PD caused by high NKCC2 activity (Fig. 3). It is possible that AVT-induced increases in IP_3_/DAG levels release the inhibition of NKCC2 in the seabream intestine. The different effects of AVT also exemplify the diversity of osmoregulatory mechanisms among teleost species [224].

The renin-angiotensin system is activated in the SW environment in teleosts [267], as indicated by elevated circulating levels of angiotensin II (Ang II) in the eel [266]. Ang II appears to inhibit NKA activity in the intestine of the eel to reduce NaCl absorption [148], and causes a reduction in plasma Na^+^ concentration in concert with an Ang II-dependent increase in NKA activity in the gills and kidneys in SW eels. Ang II also increases water and Cl^−^ absorption in intestinal sac of FW-acclimated tilapia but not in those of SW-acclimated fish [142]. It is possible that Ang II, whose receptor (AT1) is a Gαq/11-coupled GPCR, releases the inhibitory effect of NKCC2 on NaCl absorption, as discussed above for AVT. Cortisol is known to enhance NKA activity and ion/water absorption in the intestines of eels, salmonids and other model fish species [84, 151, 197, 249]. Cortisol implantation induces dose-dependent increases in NKA activity in the posterior intestine and pyloric caeca of chinook salmon [248]. However, the effect of cortisol on water transport is slow, suggesting that transcriptional activation of transporters occurs [247].

### 5.3 Hormones that affect HCO_3_^−^ transport

The effects of calciotropic hormones on HCO_3_^−^ secretion have been investigated in the teleost intestine in relation to CaCO_3_ precipitation. In teleosts, parathyroid hormone-related peptide (PTHrP) and stanniocalcin 1 (STC1) are the major hypercalcemic and hypocalcemic hormones, respectively, while calcitonin seems to have minor roles in Ca^2+^ metabolism in teleost fishes. Serosal application of pufferfish PTHrP increases ^45^Ca^2+^ influx in vitro in the seabream intestine [58], whereas salmon STC1 perfusion into the isolated cod intestine decreases Ca^2+^ influx [214]. In intestinal epithelia mounted in Ussing chambers, PTHrP decreases HCO_3_^−^ secretion, while STC1 increases HCO_3_^−^ secretion in the sea bream [59]. Interestingly, PTHrP decreases water absorption in intestinal sacs of sea bream [27]. As the in vitro experiments revealing these findings used the same fish Ringer solution on both sides of the intestinal epithelium, it would be intriguing to examine the effects of PTHrP using simulated in vivo luminal fluid on the mucosal side, in which carbonate precipitation could occur. As mentioned above, renoguanylin inhibits HCO_3_^−^ secretion in the toadfish intestine [180], while guanylin stimulates HCO_3_^−^ secretion in the eel intestine when simulated luminal fluid was used on the mucosal side [228]. In mammals, guanylin stimulates HCO_3_^−^ secretion from the duodenal epithelium in vitro [77] and in vivo [17] through activation of CFTR. Prolactin decreases HCO_3_^−^ secretion in the seabream intestine in vitro [53]. Interestingly, incubation of anterior intestinal explants with prolactin for 3 h downregulates *slc4a4* expression but not *slc26a6* or *slc26a3* expression. Expression of the two prolactin receptor genes (*prlr1* and *prlr2*) has been reported in the Mozambique tilapia intestine, and *prlr2* expression is upregulated in SW-acclimated fish [198]. A recent review has summarized the osmoregulatory action of prolactin in the gastrointestinal tracts of fishes [23].

Research on hormonal regulation of ion and water transport in the teleost intestine has focused mostly on the transcellular pathway, but paracellular transport through TJ proteins may also be regulated by hormones (see 6.1). ANP has been suggested to affect paracellular ion transport in the eel intestine [239]. In addition, several hormones have been shown to affect *cldn* expression in the Atlantic salmon [235]. Among those hormones, cortisol, growth hormone and prolactin downregulate the expression of *cldn15*, the major CLDN in the teleost intestine (see 6.1), in the anterior intestine in FW salmon. This downregulation probably regulates the changes in paracellular permeability by reorganizing intestinal tissues in preparation for downstream migration. There have been several studies on the hormonal regulation of CLDNs in the gills [116, 117]. In addition to the intestine, hormonal regulation of esophageal desalinization is also likely as mentioned above (see 2.1).

## 6. Future directions

There are a number of important questions that have not yet been answered about the role of the intestine in SW acclimation in teleost fishes. Regarding transporters, major transporters essential for NaCl and water absorption may have been identified using contemporary molecular techniques, and more will be identified in the near future using newly developed bioinformatics tools that enable detection of trace expression of genes that are important for fine-tuning of physiological responses. A more important goal is to confirm the functions of the identified genes, probably using genetically modified fishes. As discussed above, the molecular mechanisms of transport are considerably different among species, not only between euryhaline and stenohaline species but also within euryhaline species such as catadromous eels and anadromous salmonids [224]. In this regard, it is important to thoroughly establish the molecular mechanism in one species for comparison with the mechanisms in other species. The molecules include not only transporters but also TJ proteins, the latter of which constitutes the first topic of this section. As a model species, the euryhaline medaka (genus *Oryzias*) may be advantageous because a reliable genome database is publicly available [104], techniques for gene modification have been established [112], and kin species with different salinity tolerances exist [92]. The second topic of this section is Mg^2+^ and SO_4_^2−^ transport in the intestine. The intestinal epithelium has long been thought to be impermeable to these divalent ions, but they seem to be significantly able to pass through the epithelium, as indicated in eel studies [257]. The third and fourth topics have scarcely been investigated in fishes, so these topics will be discussed based on data obtained in mammals to frame future studies in fishes.

### 6.1 Paracellular route

The sheet-like polarized epithelial cells that transport ions and water are joined by TJs near the apical part of the lateral membrane, which serve as barriers for free movement of ions and water between cells [243]. Junctional proteins consist of several molecules, of which CLDNs are major constituents and play essential roles in the sealing mechanism in terms of the structure and function of TJs [78, 119]. TJs are not simple barriers for ions and water; rather, they exhibit selectivity for the substances that pass through the junctions according to their concentration gradients, and CLDNs are the major components involved in selectivity [60, 121]. The CLDN family is composed of 27 members, which are divided largely into two groups: barrier-forming CLDNs, such as CLDN1, CLDN3, CLDN4, CLDN5, CLDN6, CLDN8, CLDN12 and CLDN18, and channel-forming CLDNs, such as CLDN2, CLDN10, CLDN15, CLDN16, CLDN17 and CLDN19. The channel-forming CLDNs are further subdivided into cation-selective channel formers, such as CLDN2, CLDN10b, CLDN15, CLDN16 and CLDN19, and anion-selective channel formers, such as CLDN10a and CLDN17 [121]. CLDN10a and CLDN10b are splice variants from the same gene. Among channel-forming CLDNs, only CLDN2 serves as a paracellular water channel [175]. In leaky epithelia such as those of the intestine and renal proximal tubules, paracellular fluid transport plays a significant role in water absorption. In the anterior intestines of rodents, CLDN2 and CLDN15 are abundantly expressed and serve as routes for Na^+^ recruitment between epithelial cells for continuous activity of Na^+^-coupled nutrient absorption [229]. CLDN15 is the most studied CLDN, and its gene knockout results in an extraordinarily large intestine in terms of length and diameter (megaintestine). Recently, CLDN15 has been suggested to be a paracellular water channel in the small intestine in mammals [176].

The number of CLDN genes increased by more than double in teleost fishes via teleost-specific whole-genome duplication and tandem duplication of genes [117]. Duplicated genes are usually nonfunctionalized as pseudogenes or subjected to posttranscriptional gene silencing, but most of the CLDN genes are retained in teleosts. This retention may be related to aquatic life because the body surfaces of teleosts are exposed directly to environmental water that dissolves various substances and has an osmolality different from that of body fluids. Thus, the epithelial cells of the body surface must cope with these issues via TJs. As a result, the gills have been the major targets of CLDN research in fishes [30]. For instance, cation selectivity and salinity-dependent expression has been examined for duplicated channel-forming CLDN10s in killifish gills [147].

The expression levels of *cldn1, cldn5b, cldn7a, cldn11a, cldn11b, cldn12, cldn15, and cldn23a* have been detected by RNA-seq in the eel intestine, and *cldn7a* and *cldn15* are upregulated after SW transfer (unpublished data). Real-time qPCR has not yet been performed on *cldn*s expressed in the intestine. In the esophagus, similar sets of *cldn*s are expressed, and the number of expressed genes is much greater in the esophagus than in the intestine, illustrating the watertight nature of the esophagus (see 2.1). Among the genes, *cldn15* is expressed at the highest level, and its expression is upregulated after SW transfer in the eel intestine, as in the esophagus [227]. Of the CLDNs expressed in the eel esophagus, only CLDN15 is a cation channel type; the others are barrier-forming types. Because of the upregulation of *cldn15* after SW acclimation, paracellular Na^+^ influx through TJs could be accelerated in the esophagus after drinking of SW. In the intestine, by contrast, as the Na^+^ concentration of luminal fluid is much lower than that of extracellular fluid (Table 1), absorbed Na^+^ is recirculated  back into the luminal fluid via CLDN15 to maintain NKCC2/NCC activity. Recently, CLDN15 has been suggested to be water-permeable in mammals, but Na^+^ influx blocks water movement through the TJ protein [176]. Thus, even if teleost CLDN15 is also water-permeable, water efflux may be blocked by vigorous Na^+^ transport caused by luminal SW in the SW eel esophagus. Although Na^+^ may move through TJs, Cl^−^ may not move in parallel through the paracellular route because no candidates for anion-specific CLDNs are expressed in the eel esophagus. As CLDNs are highly diversified in teleost fishes, the selectivity of CLDNs for ions and water must be examined in diverse teleost CLDNs in the future. Hormonal regulation of paracellular transport is also an interesting topic for future studies.

In other teleost fishes, *cldn15* and *cldn25b* are expressed significantly, and their expression is upregulated after SW transfer in the intestine of the Atlantic salmon [235]. However, the expression of *cldn3* is low in this species [233]. The barrier-forming CLDN3 genes were duplicated and are expressed in different intestinal segments of the euryhaline pufferfish, *Tetraodon nigroviridis*; in addition, the expression levels decrease after transfer to SW [33]. The *cldn15a*, *cldn15b* and *cldn25* are expressed in the medaka intestine, and *cldn15b* expression is profoundly downregulated in SW [21]. As the *aqp* genes are downregulated in the medaka intestine after SW transfer [139], the decreased *cldn15* expression in SW may be related to increased water permeability of the paracellular route (see 4.2.1). Recently, Tipsmark et al. [234] suggested that CLDN15 in the medaka intestine may be water-permeable because the density of immunoreactive CLDN15 at TJs did not change in SW despite downregulation of *cldn15* transcripts.

### 6.2 Absorption of Mg^2+^ and SO_4_^2−^

It is generally accepted that Mg^2+^ and SO_4_^2−^ are hardly absorbed by the intestine in teleosts [166, 262]; thus, changes in their amounts and concentrations in the luminal fluid are sometimes used as markers to estimate the amounts of ingested SW and subsequent water absorption by the intestine, respectively. However, Mg^2+^ and SO_4_^2−^ may be absorbed significantly by the intestine because these ions are secreted into the urine at high concentrations in marine teleosts [18, 173, 257], although the fraction of renal excretion of absorbed ions appears to be small in the toadfish [69]. The Mg^2+^ transporters SLC41a1 and Cyclin M3 (Cnnm3) have been identified in the renal proximal tubules of SW-acclimated mefugu, and they have been suggested to be involved in Mg^2+^ secretion into the lumen [96, 97]. Mg^2+^ seems to be concentrated in cytoplasmic vesicles and secreted into the lumen via exocytotic delivery of vesicle contents [18]. The SO_4_^2−^ transporters SLC26a6 (apical) and SLC26a1 (basolateral) exist in renal proximal tubule cells of SW eels [256] and SW mefugu [107], and these AEs secrete SO_4_^2−^ transcellularly from the serosa to the mucosa in exchange for Cl^−^. As a result, Mg^2+^ and SO_4_^2−^ concentrations in the urine of SW-acclimated eels are 116.1 mM and 36.5 mM, respectively, higher than the concentrations in SW. In contrast, SO_4_^2−^ is actively absorbed in exchange for HCO_3_^−^ in the proximal tubules of FW eels, where apical SLC13a1 and basolateral SLC26a1 transport SO_4_^2−^ and HCO_3_^−^ in the opposite direction from that in SW eels [158]. Thus, the direction of SO_4_^2−^ transport by SLC26a1 is concentration-dependent. As Mg^2+^ is precipitated with Ca^2+^ as carbonates to decrease its concentration in the luminal fluid, SO_4_^2−^ may be absorbed to a greater extent than Mg^2+^.

Genes involved in Mg^2+^ transport are significantly expressed in the intestines of SW mefugu [96, 97]. In mammals, transient receptor potential melastatin types 6 [252] and 7 [190], Cnnm4 [276], and Mg^2+^ transporter 1 [65] are expressed in the intestine [41]. Furthermore, some TJ proteins, such as cation channel-type CLDN16 and CLDN19, are known to selectively pass Mg^2+^ through the paracellular route [118, 202]. As the Mg^2+^ concentration in the luminal fluid is extremely high (> 150 mM), the paracellular pathway needs to be taken into account. However, expression of *cldn16* and *cldn19* in the SW eel intestine is undetectable by RNA-seq. Although research on Mg^2+^ transport across the intestinal epithelium in teleost fishes is still in its infancy, this is certainly an intriguing topic for future research.

Concerning SO_4_^2−^ transport, a pair of SLC26-family transporters, apical SLC26a3/6 and basolateral SLC26a1, are substantially expressed in the intestinal epithelia of marine teleosts (see 4.4.1). These transporters have been suggested to absorb Cl^−^ and secrete HCO_3_^−^, as discussed above [69]. We also suggest that SLC26a3/6 may exchange these monovalent ions in opposing directions, i.e., via Cl^−^ secretion and HCO_3_^−^ absorption, as judged by the high HCO_3_^−^ concentration and low Cl^−^ concentration in the luminal fluid (see 4.3.1). In addition, we further suggest that this pair of SLC26 transporters may absorb SO_4_^2−^ and secrete HCO_3_^−^ or Cl^−^ (Figs. 5 and 6). This exchange is possible because of the high concentration of SO_4_^2−^ in the luminal fluid of the distal intestine (Table 1) and the high affinity of these SLC26 AEs for SO_4_^2−^ [1]. This anion exchange also increases luminal HCO_3_^−^ and Cl^−^ concentrations to facilitate carbonate precipitation and to maintain NKCC2 activity for water and NaCl absorption, respectively. It has been shown that SO_4_^2−^ is absorbed in exchange for Cl^−^ in the winter flounder intestine in vitro under high SO_4_^2−^ and low Cl^−^ in the mucosal fluid, while SO_4_^2−^ secretion occurs when both mucosal and serosal fluid contain 1 mM SO_4_^2−^ [169]. When ^35^SO_4_^2−^ is added to the environmental SW, 15% of the SO_4_^2−^ that enters the blood enters via intestinal absorption, as demonstrated by esophageal ligation in SW eels [257]. ^35^SO_4_^2−^ injected into the blood is secreted gradually into the environment; the ^35^SO_4_^2−^ appears in the urine as ions soon after injection, while it is taken up by the liver and gills. Much more ^35^S than ^35^SO_4_^2−^ is secreted slowly from the digestive tract and gills as bile compounds and mucus. Thus, more SO_4_^2−^ is absorbed by the intestine than is indicated by its urine concentration. Mefugu SLC26a6 can exchange SO_4_^2−^ with Cl^−^/HCO_3_^−^, as demonstrated via transporter expression in *Xenopus* oocytes, and the activity is dependent on the SO_4_^2−^ concentration in the medium [107]. The ability of teleost SLC26a1 to exchange SO_4_^2−^ and Cl^−^/HCO_3_^−^ needs to be verified in a similar expression system in the future. The rate of SO_4_^2−^ absorption by the intestine also needs to be calculated using an intestinal sac preparation containing different concentrations of SO_4_^2−^ in the lumen.

### 6.3 Functional coupling of transporters and enzymes (metabolon formation)

It has been suggested that in mammals, NHEs, AEs, CAII, and CAIVs form a metabolon on the apical membrane in intestinal epithelial cells to facilitate the secretion of H^+^ and HCO_3_^−^ and the absorption of Na^+^ and Cl^−^ across the apical membrane [199] (Fig. 8). Physical as well as functional association of NHE3 with CAII has been reported on the brush border membranes of renal proximal tubules, where > 65% of filtered Na^+^ is reabsorbed [120]. It has also been shown that CAII binds to the C-terminal amino acid sequence (DADD) of AE1 (SLC4a1) and other SLC4-family HCO_3_^−^ transporters (AE2, AE3, and NBCe1) to form a complex called the bicarbonate transport metabolon (Fig. 8), which maximizes transmembrane HCO_3_^−^ flux [152, 211]. In another group of AEs (the SLC26 family), SLC26a6 has such a consensus DADD sequence at the C-terminal region for CAII binding, and both physical and functional associations with the enzyme have been reported (Fig. 8). The association is disrupted by Ang II-induced PKC activation [3]. SLC26a3 does not possess the consensus sequence, and CAII has been shown to be functionally coupled but not physically coupled with SLC26a3 [210].

The physical and functional association of SLC4-family carbonate transporters with membrane-bound CAIV at the extracellular domain of transporters to form metabolons has also been reported [2, 209]. CAIV is anchored to the cell surface via a glycosylphosphatidylinositol domain and catalyzes mainly the reverse reaction to generate CO_2_ and H_2_O from concentrated HCO_3_^−^ and H^+^ in the microenvironment (Fig. 8). The generated CO_2_ can diffuse across the lipid bilayer of the plasma membrane, but the presence of AQP1 greatly accelerates its transport [171]. Water is also transported back into the cells by AQP1, which is activated by CAII bound to the C-terminal DADD motif in the cytoplasmic domain of AQP1 [251].

NHE3 and SLC26a3/6 have been suggested to interact with CFTR for regulation of mutual activity in the mammalian intestine [106] (Fig. 8). PDZ adaptor proteins such as NHERFs are scaffolding proteins that assemble membrane proteins with PDZ-binding motifs at the C-terminal region [44, 123]. Such proteins include NHE3, SLC26-family transporters and CFTR. As NHERF1 ~ NHERF4 have multiple PDZ domains within their structures, they assemble the several abovementioned transporters and anchor them to the plasma membrane (Fig. 8). They also assemble a number of kinases and cytoskeletal molecules to form a multiprotein complex that regulates transporter activity and recycling (trafficking) of endocytic vesicles carrying transporters [123]. It is also suggested that the NHERF-based complex further polymerizes to form a complex metabolon for more active  interaction among transporters. The C-terminal sequences, including the PDZ-binding motif of CFTR, are thought to determine its localization on the apical membrane [155, 217].

In intestinal epithelial cells, CFTR is coupled with SLC26 transporters through binding between its R-domain and the STAS domains of SLC26 proteins on NHERFs [115, 128] (Fig. 8). The interaction of the two transporters is usually mutually activated, resulting in increased HCO_3_^−^ secretion or Cl^−^ secretion depending upon the tissues examined [106, 115, 212]. After endogenous cAMP stimulation via hormones, anion secretion is enhanced, while Na^+^ absorption is suppressed (see 5). This is explained by inhibition of NHE3 by CFTR bound to NHERF [32]. NHE3 has a PDZ-binding motif to bind to NHERFs but has no STAS domain to bind with the CFTR R-domain. The interaction of NHE3 and SLC26a3 appears to be realized by the subcellular linkage of two NHERFs to form a complex metabolon [124]. These data regarding the interaction were obtained in a heterologous expression system using cultured cells. More recently, however, mutual regulation of CFTR, SLC26a3/6 and NHE3 has been demonstrated in vivo in in situ perfused intestines using knockout mice [204].

Based on the data from mammals mentioned above, a possible scheme for HCO_3_^−^ secretion and carbonate formation in the teleost intestine can be depicted (Fig. 8); this scheme needs to be confirmed in the future. A metabolon is proposed to exist on the apical membranes of enterocytes that consists of NHE3, SLC26a3/6, and CFTR assembled on the scaffolding protein NHERF via their PDZ-binding motifs. The *slc9a3r1* (NHERF1) is expressed in the eel intestine and upregulated in the anterior intestine after SW acclimation [228]. Cytosolic CAII and membrane-bound CAIV are connected to transporter assembly for efficient conversion of HCO_3_^−^ + H^+^ ⇌ CO_2_ + H_2_O. AQP1 also exists near the metabolon for quick recycling of CO_2_ and H_2_O across the apical membranes of enterocytes (Fig. 8). Grosell et al. [76] suggested that VHA and SLC26a6 form a metabolon on the apical membrane of the marine teleost intestinal epithelium for active HCO_3_^−^ secretion and Cl^−^ absorption. Thus, it is possible that VHA is also involved in this scheme to increase the efficiency of H^+^ acquisition and to further accelerate the conversion by CAIV and Mg/CaCO_3_ formation. The whole scheme needs to be elucidated in teleosts in the future.

### 6.4 Trafficking of transporter-tagged cytoplasmic vesicles

It is well known that AQP2 is stored on endocytic vesicles in renal collecting duct cells and inserted into the apical membrane upon stimulation by vasopressin-induced cAMP production in mammals [102]. This recruitment enables quick regulation of water reabsorption by vasopressin to cope with acute dehydration. Conversely, AQP2 on the plasma membrane is internalized via endocytic vesicles that are stored in the subapical epithelial cell layer for future recruitment. This phenomenon is called vesicle trafficking and is achieved by phosphorylation of transporters and activation of cytoskeletal molecules. In the case of AQP2, PKA is responsible for initial phosphorylation. On the other hand, the scaffolding protein NHERF with PDZ domains is connected to the actin cytoskeleton by linker proteins in the Ezrin/Radixin/Moesin (ERM) protein family and the protein complex with transporters mediates vesicle trafficking [129, 196]. Therefore, transporters with PDF-binding motifs such as CFTR, NHE3, and SLC26a6 are likely to be under the control of vesicle trafficking (Fig. 8).

Vesicle trafficking also seems to occur in teleost fishes, and the direction of trafficking is dependent upon the osmotic environment. For instance, CFTR-tagged vesicles are recruited to the basolateral membrane for Cl^−^ absorption when killifish are in FW, while the vesicles are recruited to the apical membrane for Cl^−^ secretion after acclimation to SW, as mentioned above [145]. This suggests that the direction of trafficking is relatively flexible, but such flexibility for CFTR trafificking has been demonstrated only in fishes thus far. A small population of secretory-type cells exists among intestinal epithelial cells in mammals; these cells express high levels of apical membrane CFTR [99]. Prominent CFTR-tagged vesicles are also observed beneath the apical membrane. It seems that cAMP stimulation recruits vesicles to the apical membrane and causes robust Cl^−^ and water secretion, resulting in diarrhea. In contrast, the sorting of CFTR-tagged vesicles seems to be more complex, being accomplished not by cAMP alone but by cooperation with other messengers in the killifish intestine [145].

NHEs are a group of transporters involved in intestinal Na^+^ absorption and H^+^ secretion [174]. Among the NHEs, NHE1 and NHE2 are resident-type (nontrafficking) transporters localized on the basolateral and apical membranes, respectively, in intestinal epithelial cells in mammals [280]. Thus, vesicles containing these NHEs are tagged for trafficking and fusion with the plasma membrane soon after protein synthesis. On the other hand, NHE3-containing cytoplasmic vesicles are tagged and inserted into the apical plasma membrane upon stimulation by intracellular messengers [280]. Since *nhe2* and *nhe3* are expressed in significant amounts in the esophagus and intestine in eels and other teleosts, it would be interesting to investigate whether a similar difference in the regulation of trafficking also exists among NHEs in teleost fishes.

## Conclusions

1. Anguillid eels are extraordinarily euryhaline teleosts and offer many advantages for research on the role of the digestive tract in SW acclimation. Although eels are now endangered species, research in cultured eels will enable great progress to be made toward understanding the mechanisms of SW acclimation in the future.
2. The key processes in eels (and marine teleosts) that enable water gain after SW drinking are desalinization by the esophagus without osmotic water loss, bicarbonate secretion for Mg/CaCO_3_ precipitation in the intestine, and efficient water/NaCl absorption by the intestine using unique transporters.
3. The major transporters responsible for such processes have been illuminated in eels and other marine teleosts. As transcriptomic data on the digestive tracts of eels after transfer from FW to SW are available to the public (DDBJ accession number: DRA004258), genes that are involved in fine-tuning of transporter function will be elucidated in the near future.
4. Several biological processes in the digestive tract that have not received sufficient attention but need to be investigated are suggested for future studies.

## Supplementary Information

**Additional file 1: Supplementary Table 1.** Primer sequences for quantitative real time PCR

## Data Availability

The RNA-seq datasets used in this review are available at the DDBJ homepage (https://www.ddbj.nig.ac.jp/index-e.html) under accession number DRA004258.

## Abbreviations

AC: Adenylyl cyclase
AE: Anion exchanger
ANP: Atrial natriuretic peptide
AQP: Aquaporin
BNP: B-type natriuretic peptide
CA: Carbonic anhydrase
CFTR: Cystic fibrosis transmembrane conductance regulator
ClC: Cl^−^ channel
Cnnm: Cyclin M
CNP: C-type natriuretic peptide
DIDS: 4,4′-diisothiocyano-2,2′-disulfonic acid
DMA: Dimethyl amiloride
DMSO: Dimethyl sulfoxide
DNDS: 4,4′-dinitrostilbene-2,2′-disulfonic acid
DPC: Diphenylamine-2-carboxylic acid
DRA: Downregulated in adenoma
EIPA: Ethylisopropyl amiloride
ENaC: Epithelial Na^+^ channel
FW: Freshwater
GC: Guanylyl cyclase
HCTZ: Hydrochlorothiazide
Isc: Short-circuit current
KCC: K^+^-Cl^−^ cotransporter
LIS: Lateral interspace
NBC: Na^+^-HCO_3_^−^ cotransporter
NCC: Na^+^-Cl^−^ cotransporter
NHE: Na^+^/H^+^ exchanger
NHERF: NHE regulatory factor
NKA: Na^+^/K^+^-ATPase
NKCC: Na^+^-K^+^-2Cl^−^ cotransporter
NPPB: 5-nitro-2-(3-phenylpropylamino)-benzoate
PAT1: Putative anion transporter 1
PD: Potential difference
PDZ: Postsynaptic density-95/discs large/zonula occludens-1
ROMK: Renal outer medullary potassium channel
SAT1: Sulfate anion transporter 1
SLC: Solute carrier
SW: Seawater
STAS: Sulfate transporter antisigma
TAL: Thick ascending limb of Henle’s loop
VHA: Vacuolar-type H^+^-ATPase
VNP: Ventricular natriuretic peptide
